# The WBC/HDL ratio outperforms other lipid profiles in predicting mortality among ischemic stroke patients: a retrospective cohort study using MIMIC-IV data

**DOI:** 10.3389/fneur.2025.1534381

**Published:** 2025-04-30

**Authors:** Li Zou, Dong Sun, Lei Zhang, Yu Xie, Renwei Zhang, Huagang Li, Bitang Dan, Yumin Liu, Bin Mei

**Affiliations:** Department of Neurology, Zhongnan Hospital of Wuhan University, Wuhan, Hubei, China

**Keywords:** lipid profiles, white blood cell to HDL ratio, stroke mortality, inflammation, MIMIC-IV database

## Abstract

**Objective:**

To assess the prognostic value of lipid profiles and their ratios, particularly the white blood cell to high-density lipoprotein (WBC/HDL) ratio, for predicting 28-day and 1-year all-cause mortality in ischemic stroke patients admitted to the ICU.

**Methods:**

A retrospective cohort study was conducted using the MIMIC-IV ICU database, including 2,894 ischemic stroke patients. Lipid profiles—including total cholesterol, triglycerides, low-density lipoprotein, and high-density lipoprotein—and derived ratios were analyzed. Associations with mortality were assessed using Cox proportional hazards models adjusted for demographic and clinical factors. Restricted cubic spline and Kaplan–Meier survival analyses were utilized to explore the relationship between the WBC/HDL ratio and mortality risk.

**Results:**

Traditional lipid profiles and their ratios were not significantly associated with 28-day or 1-year mortality. Conversely, an elevated WBC/HDL ratio was independently associated with increased mortality risk at both 28 days (hazard ratio: 2.198; 95% confidence interval: 1.864–3.225) and 1 year (hazard ratio: 3.163; 95% confidence interval: 2.947–3.334). Restricted cubic spline analysis demonstrated a linear relationship between the WBC/HDL ratio and mortality risk, while Kaplan–Meier analyses indicated significantly poorer survival outcomes for patients with higher WBC/HDL ratios.

**Interpretation:**

The WBC/HDL ratio is a superior prognostic marker for mortality in ischemic stroke patients admitted to the ICU, outperforming traditional lipid profiles. Incorporating this measure into clinical practice may enhance early risk stratification and guide targeted interventions.

## 1 Introduction

Ischemic stroke (IS) is a leading cause of disability and mortality worldwide, imposing a significant burden on patients, families, and healthcare systems (1). Thrombolysis and endovascular embolectomy have significantly improved the prognosis of ischemic stroke patients; however, many patients continue to experience poor outcomes, particularly those requiring intensive care unit (ICU) admission due to severe neurological deficits or medical complications (2). Identifying high-risk patients early remains essential to enhance postoperative management, optimize therapeutic strategies, and allocate resources effectively within the ICU setting.

Lipid metabolism and inflammation are pivotal in the pathogenesis and progression of IS. Dyslipidemia contributes to atherosclerosis and thrombosis, fundamental mechanisms underlying ischemic events (3). Elevated levels of low-density lipoprotein (LDL) and triglycerides (TG), along with decreased high-density lipoprotein (HDL), have been associated with an increased risk of stroke occurrence (4). In parallel, systemic inflammation plays a critical role in neuronal injury and post-stroke recovery, with elevated inflammatory markers correlating with worse outcomes (5, 6).

Given the interconnected roles of lipid metabolism and inflammation in IS, ratios that integrate these parameters have been proposed as comprehensive prognostic markers. Ratios like LDL/HDL, TC/HDL (total cholesterol to HDL ratio), and TG/HDL aim to reflect the balance between pro-atherogenic lipids and protective HDL (7). The white blood cell to HDL ratio (WBC/HDL), in particular, combines systemic inflammation with lipid status, potentially offering a more holistic assessment of risk (8). Prior studies suggest that these composite ratios may hold stronger prognostic value than individual lipid measures in cardiovascular disease (9, 10), yet their predictive utility in ICU-admitted IS patients remains uncertain. Therefore, critical illness and the body’s acute response can change lipid levels and inflammation markers, which may influence their usefulness for predicting outcomes (11, 12). Understanding whether traditional lipid profiles and their ratios can still reliably predict outcomes in this setting is crucial for identifying effective markers to assess risk and guide treatment in the ICU.

Prior studies suggest that these composite ratios may hold stronger prognostic value than individual lipid measures in cardiovascular disease (9, 10), yet their predictive utility in ICU-admitted IS patients remains uncertain. This study therefore aimed to evaluate two critical questions using the MIMIC-IV database (13): first, whether traditional lipid ratios (LDL/HDL, TC/HDL, TG/HDL) retain prognostic value in critically ill stroke patients experiencing acute metabolic disturbances; and second, whether novel ratios integrating systemic inflammation with lipid profiles (e.g., WBC/HDL) could provide enhanced risk stratification in this population. Through this investigation, we sought to establish evidence-based guidance for prognostic marker selection in neurocritical care settings.

## 2 Methods

### 2.1 Data source

This study utilized data from the MIMIC-IV database (version 2.2), is a comprehensive, publicly accessible database that contains de-identified health-related information from over 70,000 ICU admissions at Beth Israel Deaconess Medical Center in Boston, Massachusetts, USA, spanning the years 2008 to 2019. The database was developed by the Laboratory for Computational Physiology at the Massachusetts Institute of Technology. To ensure patient confidentiality, all personal identifiers were removed, and the data were fully anonymized, eliminating the need for individual patient consent or institutional review board approval. Access to the database was granted through the PhysioNet platform^1^ after the author Li Zou (ID: 13349610) completed the required training courses from the Collaborative Institutional Training Initiative (CITI) program on “Conflict of Interest” and “Data or Specimens Only Research.”

### 2.2 Population selection and outcomes

**Figure 1:**
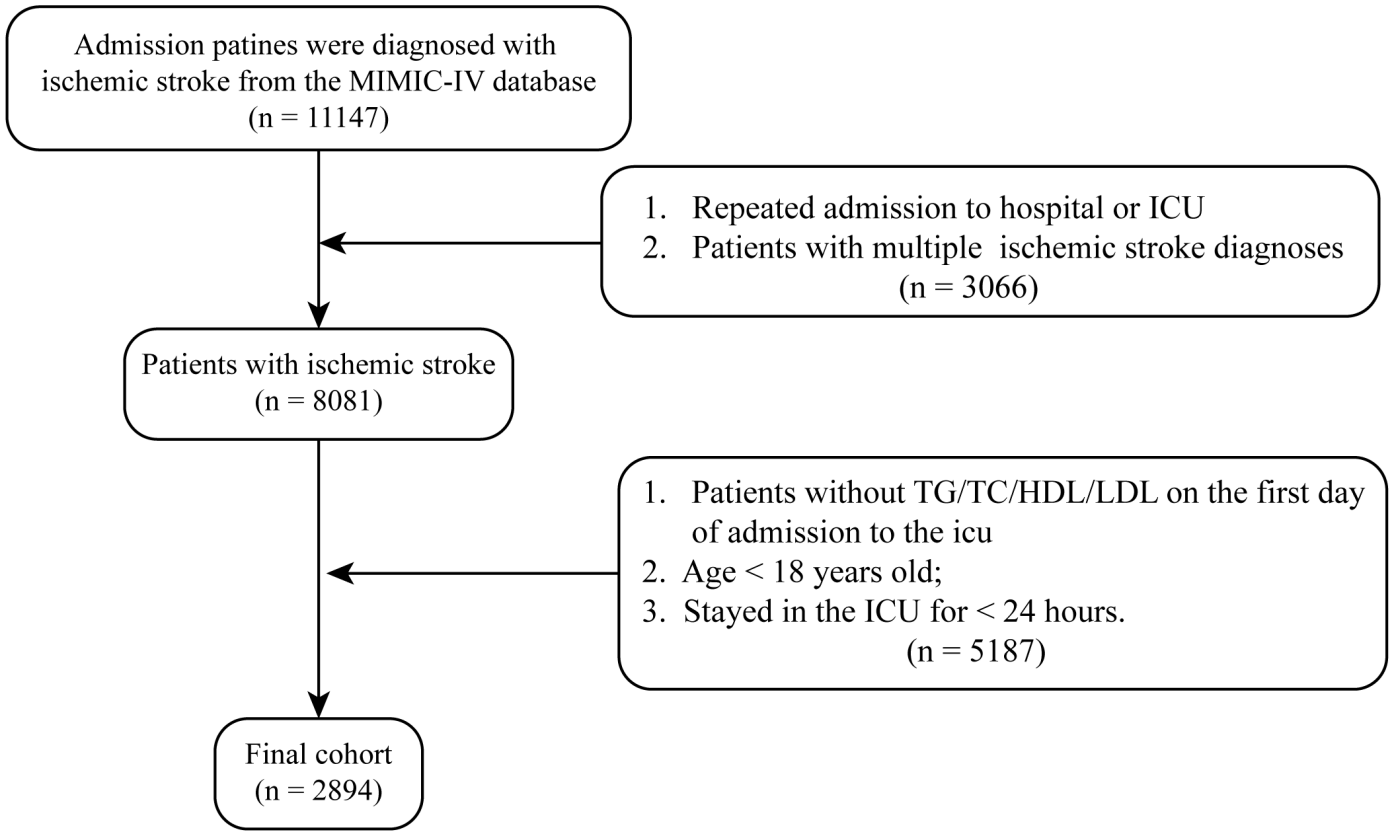
The study flowchart for ischemic stroke patients from MIMIC-IV database. Flowchart illustrating the selection process of ischemic stroke patients from the MIMIC-IV database. A total of 8,081 patients with a diagnosis of ischemic stroke were initially identified. After applying exclusion criteria—including missing key laboratory data, age under 18 years, and stayed in the ICU over 24 h—a final cohort of 2,894 patients was included in the analysis.

This study retrospectively included patients admitted to the ICU with a diagnosis of ischemic stroke. The inclusion criteria were: (1) age above 18 years, (2) a diagnosis of ischemic stroke identified using ICD-9 and ICD-10 codes, and (3) ICU stay duration of at least 24 h. Patients with multiple ICU admissions were analyzed only for their first ICU admission, and patients with missing critical data were excluded. Ultimately, 2,894 patients were included in this study (Figure 1).

### 2.3 Data extraction

This study focused on assessing the impact of lipid profiles and their relative ratios on the one-year survival rate of patients with ischemic stroke admitted to the ICU. The primary exposure variables included traditional lipid parameters—TC, TG, LDL, and HDL. To evaluate the prognostic significance of these biomarkers and their interplay with lipid metabolism and inflammation, we calculated several ratios: TC/HDL, TG/HDL, LDL-C/HDL, and WBC/HDL. These values were derived from measurements taken at the time of each patient’s initial ICU admission.

The primary outcome was all-cause mortality within one year following ICU admission. Mortality data were collected at multiple time intervals—7 days, 14 days, 28 days, 90 days, and 1 year post-admission—to facilitate both short-term and long-term survival analyses. Secondary outcomes included the length of ICU stay and total hospital stay.

To adjust for potential confounding factors, we collected comprehensive data on demographic characteristics (such as age and sex), clinical severity scores (including the Charlson Comorbidity Index [CCI], and Sequential Organ Failure Assessment [SOFA] score), laboratory findings, and therapeutic interventions (e.g., use of vasopressors). A detailed list of all variables included in the analysis is provided in Table 1.

**Table 1:** Covariates extracted in detail from the MIMIC-IV database.

Items	Composition
Demographic Details	Ethnicity, age, gender, height, weight
Vital Signs	Systolic and diastolic blood pressure (SBP & DBP), mean blood pressure (MBP), heart rate (HR), respiratory rate, Oxygen saturation (SpO_2_)
Scales	Glasgow coma scale (GCS), Sequential Organ Failure Assessment Score (SOFA score), Simplified Acute Physiology Score II (SAPS II), Acute Physiology Score III (APS III), Oxford Acute Severity of Illness Score (OASIS score), Systemic Inflammatory Response Syndrome Score (SIRS score)
Comorbid Conditions	Hypertension, acute myocardial infarction (AMI), heart failure, cardiac arrhythmias, diabetes, peripheral vascular disease (PVD), chronic pulmonary disease, respiratory failure, ventilator-associated pneumonia (VAP), chronic kidney disease (CKD), hyperlipidemia, malignancy, renal failure, sepsis, liver disease, Carlson comorbidity index (CCI)
Laboratory Parameters	Red and white blood cell counts, platelet counts, Hemoglobin (Hb), sodium, potassium, blood urea nitrogen (BUN), creatinine, albumin, Alanine Aminotransferases (ALT), Aspartate Aminotransferase (AST), Lactate Dehydrogenase (LDH), triglyceride (TC), triglyceride (TG), High-Density Lipoprotein (HDL), Low-Density Lipoprotein (LDL), anion gap, lactate, D-dimer, fibrinogen, International Normalized Ratio (INR), Prothrombin Time (PT), Activated Partial Thromboplastin Time (APTT), C-Reactive Protein (CRP), globulin, total protein, thrombin, Homocysteine (Hcy)
Clinical Treatment	Vasopressors, oxygen, Continuous Renal Replacement Therapy (CRRT), thrombolysis, thrombectomy
Clinical Outcomes	ICU_Stays, Hospital_Stays, ICU mortality, In-hospital mortality, 7-day mortality, 14-day mortality, 21-day mortality, 28-day mortality, 90-day mortality, 1-year mortality

Data extraction was performed using Structured Query Language (SQL) queries within the PostgreSQL database management system (version 2.7.3), managed through pgAdmin4 (version 8.6). Rigorous data validation procedures were implemented to ensure the accuracy and consistency of the extracted information.

### 2.4 Management of missing data and outliers

To ensure the robustness of our analysis, we carefully addressed missing data and outliers. Variables with more than 15% missing values, such as lymphocyte count, monocyte count, neutrophil count, globulin, total protein, thrombin, Homocysteine (Hcy) and C-reactive protein (CRP), were excluded to minimize potential bias introduced by imputation. For variables with less than 15% missing data—including blood urea nitrogen (BUN), Sodium, Creatinine and Albumin—we employed multiple imputation using chained equations to create a complete dataset for analysis. This method preserves the relationships between variables by generating plausible values based on the observed data (14). This approach ensured the integrity of the data and maintained the reliability of subsequent analyses.

### 2.5 Follow-up and endpoints

The study’s follow-up period spanned 1 year from the date of ICU admission. We focused on all-cause mortality at specific intervals: 7 days, 14 days, 21 days, 28 days, 90 days, and 1 year post-admission, to capture both short-term and long-term outcomes. Mortality data were extracted from the hospital’s electronic health records, ensuring comprehensive capture of in-hospital deaths and post-discharge mortality through linkage with national death registries when available.

The primary endpoint was defined as all-cause mortality within 1 year of ICU admission. Survival time was calculated from the date of ICU admission to the date of death or the end of the one-year follow-up period, whichever occurred first. Patients who survived beyond the follow-up period were censored at 1 year. The diagnostic criteria and definitions of outcomes were based on standardized hospital protocols, ensuring consistency across all cases.

### 2.6 Statistical analysis

We used descriptive statistics to summarize the baseline characteristics of the patients. Continuous variables were tested for normality, with normally distributed variables presented as mean ± standard deviation (SD) and non-normally distributed variables as median and interquartile range (IQR). Categorical variables are shown as counts and percentages. Group comparisons for continuous variables utilized the Student’s t-test or Mann–Whitney U test, depending on distribution, and chi-square or Fisher’s exact tests were applied for categorical variables. The lipid ratios of interest—TC/HDL, TG/HDL, LDL-C/HDL, and WBC/HDL—were categorized by median values or clinically established cut-off points. We conducted univariate Cox proportional hazards regression analyses to identify potential predictors of mortality at various time points (7-day, 14-day, 21-day, 28-day, 90-day, and 1-year). Variables deemed significant in univariate analyses or those with clinical relevance were included in multivariable Cox regression models to determine independent predictors of mortality across these intervals. In these models, we adjusted for potential confounders, including demographic factors and clinical severity scores, to isolate the independent effects of WBC/HDL and other lipid ratios. To evaluate the prognostic value of the WBC/HDL ratio and other lipid ratios, we performed receiver operating characteristic (ROC) curve analyses and calculated the area under the curve (AUC) to assess predictive accuracy for each mortality time point. The optimal cutoff value for WBC/HDL was determined using the Youden index. Additionally, restricted cubic spline (RCS) regression was used to explore non-linear associations between the WBC/HDL ratio and mortality risk over time. Subgroup analyses were conducted to assess the consistency of the association between the WBC/HDL ratio and mortality across various patient populations. Subgroups included clinically significant factors such as gender, platelet count, and Charlson Comorbidity Index (CCI) levels. Survival distributions for these subgroups were illustrated using Kaplan–Meier survival curves, and differences were evaluated with the log-rank test. All statistical analyses were performed using R software (version 4.3.2) and SPSS software (version 29.0). A *p*-value of less than 0.05 was considered statistically significant for all hypothesis tests.

## 3 Results

### 3.1 Baseline characteristics of the study population

Table 2 presents the baseline characteristics of the 2,894 ischemic stroke patients included in the study, with a median age of 73 years (interquartile range [IQR]: 63–83 years) and 51.07% male. The overall 28-day and 1-year mortality rates were approximately 15.5 and 27.8%, respectively. Significant differences were observed between survivors and non-survivors at both endpoints. Non-survivors were significantly older than survivors at both the 28-day (median age 80 vs. 72 years; *p* < 0.001) and 1-year (median age 79 vs. 70 years; *p* < 0.001) marks. In general indicators, non-survivors also weighed more than survivors at 28-day (median, 76.6 kg vs. 72.4 kg; *p* < 0.001) and 1-year (median, 77.8 kg vs. 72.2 kg; *p* < 0.001). They also had higher prevalence of comorbidities such as heart failure (28-day: 30.79% vs. 25.93%, *p* = 0.007; 1-year: 33.87% vs. 23.75%, *p* < 0.001), respiratory failure (28-day: 32.29% vs. 19.67%, *p* < 0.001), and malignancy (28-day: 13.81% vs. 6.91%, *p* < 0.001). Non-survivors exhibited higher CCI scores (28-day median: 7 vs. 6; 1-year median: 7 vs. 5; both *p* < 0.001) and elevated clinical severity scores including SOFA score, SAPS II, and APS III (all *p* < 0.001), indicating greater disease severity.

**Table 2:** Baseline characteristics of ischemic stroke patients stratified by 28-day and 1-year survival status.

Variable	Total	28-Day			1-Year		
		Survivors	non-Survivors	*p*	Survivors	non-Survivors	*p*
	(*n* = 2,894)	(*n* = 2,445)	(*n* = 449)		(*n* = 2,088)	(*n* = 806)	
Age, median (IQR)	73 (63, 83)	72 (61, 82)	80 (71, 88)	<0.001	70 (60, 80)	79 (70, 87)	<0.001
Gender: male, n (%)	1,478 (51.07)	1,272 (52.02)	206 (45.88)	0.019	1,105 (52.92)	373 (46.28)	0.001
Ethnicity: White, n (%)	1,898 (65.58)	1,615 (66.05)	283 (63.03)	0.214	1,381 (66.14)	517 (64.14)	0.315
Asian, n (%)	90 (3.11)	76 (3.11)	14 (3.12)	1.000	67 (3.21)	23 (2.85)	0.720
Black and Hispanic/Latino, n (%)	464 (16.03)	409 (16.73)	55 (12.25)	0.017	337 (16.14)	127 (15.76)	0.821
Other, n (%)	442 (15.27)	345 (14.11)	97 (21.60)	<0.001	303 (14.51)	139 (17.25)	0.073
Height, median (IQR), cm	167.64 (160.02, 175.26)	167.6 (161.29, 176.53)	166.4 (158.75, 175)	<0.001	168.0 (162.56, 177.8)	165.1 (160, 175)	<0.001
Weight, median (IQR), kg	76.5 (64.33, 90.00)	76.6 (65.5, 91)	72.4 (59, 82.3)	<0.001	77.8 (66.7, 92.3)	72.2 (59.93, 82.83)	<0.001
MBP, median (IQR), mmHg	82 (74, 92)	82 (74, 92)	82 (74, 92)	0.916	82 (75, 92)	80 (72, 89)	<0.001
SBP, median (IQR), mmHg	127 (114, 142)	126 (114, 141)	129 (114, 145)	0.108	127 (114, 142)	126 (112, 142)	0.081
DBP, median (IQR), mmHg	65 (57, 74)	65 (57, 75)	64 (58, 74)	0.568	66 (57, 76)	63 (57, 72)	<0.001
Heart Rate, median (IQR), bpm	80 (71, 90)	78 (69, 88)	84 (74, 95)	<0.001	78 (69, 88)	84 (73, 95)	<0.001
Respire Rate, median (IQR), bpm	19 (17, 21)	18 (16, 20)	19 (17, 22)	<0.001	18 (17, 20)	20 (17, 22)	<0.001
SpO_2_, median (IQR), %	97 (95, 98)	97 (96, 98)	97 (96, 99)	0.028	97 (96, 98)	97 (96, 99)	0.02
GCS, median (IQR)	15 (14, 15)	15 (14, 15)	15 (13, 15)	<0.001	15 (14, 15)	13 (13, 15)	<0.001
Sofa score, median (IQR)	3 (2, 5)	3 (2, 5)	5 (3, 7)	<0.001	3 (2, 5)	4 (3, 7)	<0.001
SAPS II, median (IQR)	35 (27, 43)	33 (26, 41)	42 (35, 52)	<0.001	31.5 (25, 39)	41 (34, 50)	<0.001
APS III, median (IQR)	38 (28, 51)	36 (27, 49)	50 (37, 65)	<0.001	35 (26, 46)	48.5 (36, 62)	<0.001
OASIS, median (IQR)	31 (25, 37)	30 (25, 35)	37 (32, 42)	<0.001	29 (24, 35)	36 (30, 41)	<0.001
SIRS, median (IQR)	2 (2, 3)	2 (2, 3)	3 (2, 3)	<0.001	2 (2, 3)	3 (2, 3)	<0.001
Hypertension, n (%)	2,424 (83.76)	2,037 (83.31)	387 (86.19)	0.147	1,755 (84.05)	699 (83.00)	0.528
Diabetes, n (%)	1,028 (35.52)	880 (35.99)	148 (32.96)	0.238	735 (35.20)	293 (36.35)	0.288
Acute Myocardial infarct, n (%)	473 (16.34)	391 (15.99)	82 (18.26)	0.259	328 (15.71)	145 (17.99)	0.152
Heart failure, n (%)	769 (26.57)	626 (25.93)	117 (30.79)	0.007	496 (23.75)	273 (33.87)	<0.001
Peripheral vascular disease, n (%)	418 (14.44)	364 (14.88)	54 (12.03)	0.131	308 (14.75)	110 (13.65)	0.485
Chronic pulmonary disease, n (%)	593 (20.49)	500 (20.45)	93 (20.71)	0.949	405 (19.40)	188 (23.32)	0.022
Respiratory failure, n (%)	626 (21.63)	481 (19.67)	145 (32.29)	<0.001	390 (18.68)	236 (29.28)	<0.001
VAP, n (%)	143 (4.94)	116 (4.74)	27 (6.01)	0.306	86 (4.12)	57 (7.07)	0.001
CKD, n (%)	612 (21.15)	504 (20.61)	108 (24.05)	0.115	400 (19.16)	212 (26.30)	<0.001
Renal failure, n (%)	320 (11.06)	226 (9.24)	94 (20.94)	<0.001	170 (8.14)	150 (18.61)	<0.001
Hyperlipidemia, n (%)	1,409 (48.69)	1,188 (48.59)	221 (49.22)	0.846	1,019 (48.80)	390 (48.39)	0.873
Malignancy, n (%)	231 (7.98)	169 (6.91)	62 (13.81)	<0.001	112 (5.36)	119 (14.76)	<0.001
Liver disease, n (%)	257 (8.88)	219 (8.96)	38 (8.46)	0.804	173 (8.29)	84 (10.42)	0.082
Sepsis: = 1, n (%)	525 (18.14)	491 (20.08)	34 (7.57)	<0.001	441 (21.12)	84 (10.42)	<0.001
Sepsis: = 2, n (%)	1,031 (35.63)	898 (36.73)	133 (29.62)	0.004	763 (36.54)	268 (33.25)	0.101
Sepsis: = 3, n (%)	938 (32.41)	756 (30.92)	182 (40.53)	<0.001	631 (30.22)	307 (38.09)	<0.001
Sepsis: = 4, n (%)	323 (11.16)	226 (9.24)	97 (21.60)	<0.001	189 (9.05)	134 (16.63)	<0.001
CCI, median (IQR)	6 (4, 8)	6 (4, 7)	7 (6, 9)	<0.001	5 (4, 7)	7 (5, 9)	<0.001
Vasopressors, n (%)	131 (4.53)	84 (3.44)	47 (10.47)	<0.001	65 (3.11)	66 (8.19)	<0.001
Oxygen, n (%)	2,054 (70.97)	1,781 (72.84)	273 (60.80)	<0.001	1,502 (71.93)	552 (68.49)	0.074
CRRT, n (%)	79 (2.73)	58 (2.37)	21 (4.68)	0.009	46 (2.92)	28 (3.47)	0.008
Thrombolysis, n (%)	89 (3.08)	79 (3.23)	10 (2.23)	0.325	61 (2.92)	28 (3.47)	0.514
Thrombectomy, n (%)	267 (9.23)	226 (9.24)	41 (9.13)	1.000	198 (9.48)	69 (8.56)	0.485
RBC, median (IQR), 10^9/L	3.83 (3.29, 4.31)	3.83 (3.31, 4.33)	3.75 (3.17, 4.17)	<0.001	3.84 (3.39, 4.38)	3.66 (3.11, 4.10)	<0.001
WBC, median (IQR), 10^9/L	8.7 (6.9, 11.1)	8.7 (6.8, 10.8)	9.7 (7.5, 13.8)	<0.001	8.7 (6.8, 10.7)	8.9 (7.1, 12.5)	<0.001
Platelet, median (IQR), 10^9/L	224 (174, 281)	224 (177, 282)	220 (161, 276)	0.016	224 (178, 281)	224 (161, 282)	0.052
Hb, median (IQR), g/L	11.3 (9.73, 12.80)	11.3 (9.8, 12.9)	11.1 (9.5, 12.4)	<0.001	11.4 (10.0, 13.1)	10.8 (9.1, 12.3)	<0.001
Sodium, median (IQR), mmol/L	140 (137, 142)	139 (137, 142)	140 (137, 143)	<0.001	139 (137, 142)	140 (137, 143)	<0.001
Potassium, median (IQR), mmol/L	4.1 (3.8, 4.4)	4.1 (3.8, 4.4)	4.0 (3.7, 4.4)	0.025	4.1 (3.8, 4.4)	4.0 (3.7, 4.4)	0.031
BUN, median (IQR), mg/dL	19 (14, 29)	19 (14, 28)	23 (16, 35)	<0.001	18 (13, 26)	23 (15.25, 35)	<0.001
Creatinine, median (IQR), mg/24 h	1 (0.7, 1.3)	1.0 (0.7, 1.3)	1.1 (0.8, 1.5)	<0.001	1 (0.7, 1.3)	1 (0.8, 1.5)	<0.001
Albumin, median (IQR), g/dL	4.1 (3.7, 4.4)	4.1 (3.8, 4.4)	3.9 (3.5, 4.3)	<0.001	4.2 (3.8, 4.5)	3.9 (3.5, 4.3)	<0.001
ALT, median (IQR), U/L	33 (20, 66)	33 (20, 66)	29 (17, 66)	0.007	33 (20, 65)	33 (19, 75)	0.629
AST, median (IQR), U/L	41 (26, 81)	41 (26, 81)	41 (26, 84)	0.456	40 (25, 76)	47 (28, 91)	<0.001
LDH, median (IQR), U/L	278 (207,402)	270 (201, 396)	310 (236, 438)	<0.001	260 (195, 379)	317 (237, 449)	<0.001
TC, median (IQR), mg/dL	170.5 (138, 208)	174 (140, 211)	156 (129, 198)	<0.001	175 (141, 212)	159 (130, 200)	<0.001
TG, median (IQR), mg/dL	126 (90, 188)	129 (93, 192)	115 (81, 168)	<0.001	130.5 (93, 195)	118 (85, 170)	<0.001
HDL, median (IQR), mg/dL	49 (38. 62)	49 (39, 63)	48 (35, 60)	0.014	49 (39, 63)	48 (36, 60)	0.0058
LDL, median (IQR), mg/dL	94 (68, 123)	95 (69, 125)	86 (61, 114)	<0.001	96 (70, 126)	86 (62, 114)	<0.001
Anion gap, median (IQR), mmol/L	14 (12, 16)	14 (12, 16)	15 (12, 17)	<0.001	14 (12, 16)	15 (12, 17)	<0.001
Lactate, median (IQR), mmol/L	2.4 (1.6, 3.5)	2.3 (1.6, 3.5)	2.5 (1.7, 3.8)	0.006	2.3 (1.6, 3.4)	2.6 (1.8, 3.9)	<0.001
PT, median (IQR), s	12.9 (11.7, 15.1)	12.9 (11.6, 15.1)	13.1 (11.9, 15.6)	0.058	12.8 (11.6, 14.9)	13.4 (12, 15.9)	<0.001
APTT, median (IQR), s	30.7 (27.2, 40.5)	30.8 (27.4, 40.8)	30.1 (26.6, 37.8)	0.008	30.7 (27.3, 40.35)	30.85 (27.03, 40.5)	0.708
INR, median (IQR)	1.2 (1.1, 1.4)	1.2 (1.1, 1.4)	1.2 (1.1, 1.4)	0.052	1.2 (1, 1.4)	1.2 (1.1, 1.5)	<0.001
TG/HDL, median (IQR)	2.66 (1.67, 4.43)	2.71 (1.69, 4.44)	2.41 (1.54, 4.22)	0.029	2.72 (1.70, 4.46)	2.50 (1.57, 4.23)	0.048
TC/HDL, median (IQR)	3.42 (2.70, 4.42)	3.43 (2.72, 4.43)	3.32 (2.66, 4.28)	0.211	3.34 (2.72, 4.48)	3.37 (2.67, 4.27)	0.076
LDL/HDL, median (IQR)	1.89 (1.35, 2.61)	1.91 (1.36, 2.63)	1.85 (1.30, 2.49)	0.175	1.92 (1.38, 2.66)	1.85 (1.29, 2.45)	0.022
WBC/HDL, median (IQR)	0.18 (0.13, 0.27)	0.18 (0.12, 0.26)	0.22 (0.13, 0.33)	<0.001	0.17 (0.12, 0.25)	0.21 (0.13, 0.30)	<0.001

SBP, Systolic Blood Pressure; DBP, Diastolic Blood Pressure; MBP, Mean Blood Pressure; HR, Heart Rate; SpO_2_, Peripheral Capillary Oxygen Saturation; RR, Respiratory Rate; GCS, Glasgow Coma Scale; SOFA, Sequential Organ Failure Assessment; SAPS II, Simplified Acute Physiology Score II; APS III, Acute Physiology Score III; OASIS, Oxford Acute Severity of Illness Score; SIRS, Systemic Inflammatory Response Syndrome; VAP, Ventilator-Associated Pneumonia; CKD, Chronic Kidney Disease; CCI, Charlson comorbidity index; CRRT, Continuous Renal Replacement Therapy; RBC, Red Blood Cell; WBC, White Blood Cell; Hb, Hemoglobin; BUN, Blood Urea Nitrogen; ALT, Alanine Aminotransferase; AST, Aspartate Aminotransferase; LDH, Lactate Dehydrogenase; TC, Total Cholesterol; TG, Triglycerides; HDL, High-Density Lipoprotein; LDL, Low-Density Lipoprotein; CRP, C-Reactive Protein; PT, Prothrombin Time; APTT, Activated Partial Thromboplastin Time; INR, International Normalized Ratio; PLR, Platelet-to-Lymphocyte Ratio; NLR, Neutrophil-to-Lymphocyte Ratio; IQR, Interquartile Range; TG/HDL, Triglyceride-to-High-Density Lipoprotein Ratio; TC/HDL, Total Cholesterol-to-High-Density Lipoprotein Ratio; LDL/HDL, Low-Density Lipoprotein-to-High-Density Lipoprotein Ratio; WBC/HDL, White Blood Cell-to-High-Density Lipoprotein Ratio.

Laboratory markers revealed that non-survivors had significantly higher WBC counts, lactate dehydrogenase (LDH), lactate levels, and WBC/HDL ratios (all *p* < 0.05), along with lower HDL levels and platelet counts (both *p* < 0.05). These patterns were consistent at both the 28-day and 1-year endpoints. Notably, the WBC/HDL ratio emerged as a significant marker for mortality in IS patients, with non-survivors consistently exhibiting higher ratios than survivors at both time points. This suggests that the WBC/HDL ratio may serve as an important prognostic indicator for mortality risk in this patient population.

### 3.2 Univariate and multivariable Cox regression analyses for mortality prediction

In the univariate Cox regression analyses (Table 3), several clinical and lipid-related factors—including age, SOFA score, and lipid ratios such as WBC/HDL—were significantly associated with both 28-day and 1-year all-cause mortality. Notably, the WBC/HDL ratio emerged as a strong predictor, with unadjusted hazard ratios (HRs) of 2.947 (95% CI: 2.203–3.942; *p* < 0.001) for 28-day mortality and 3.163 (95% CI: 1.945–3.334; *p* < 0.001) for 1-year mortality. While TG and HDL cholesterol levels were also significant predictors, their HRs were comparatively modest.

**Table 3:** Univariate Cox regression analysis of lipid profiles and their ratios associated with 28-day and 1-year all-cause mortality in patients with ischemic stroke.

Variable	28-Day			1-Year		
	HR	95%CI	*p*	HR	95%CI	*p*
Age	1.042	1.034–1.05	**<0.001**	1.040	1.034–1.046	**<0.001**
Gender	0.787	0.654–0.948	**0.012**	1.042	0.692–0.912	**0.001**
Ethnicity: refer. White						
Asian	1.062	0.587–1.046	0.817	0.826	0.627–1.445	0.817
Black and Hispanic/Latino	0.784	0.815–1.202	0.098	0.657	0.815–1.202	0.918
Other	1.542	1.225–1.943	**<0.001**	1.279	1.007–1.465	**0.042**
Height	0.984	0.975–0.992	**<0.001**	0.985	0.979–0.991	**<0.001**
Weight	0.982	0.977–0.988	**<0.001**	0.982	0.979–0.986	**<0.001**
MBP	0.998	0.991–1.005	0.600	0.987	0.981–0.992	**<0.001**
SBP	1.004	0.999–1.009	0.112	0.997	0.994–1.001	0.170
DBP	0.996	0.989–1.003	0.278	0.986	0.981–0.992	**<0.001**
Heart Rate	1.021	1.015–1.027	**<0.001**	1.019	1.015–1.024	**<0.001**
Respire Rate	1.104	1.079–1.129	**<0.001**	1.093	1.074–1.113	**<0.001**
SpO_2_	1.052	0.999–1.108	0.055	1.042	1.003–1.083	**0.036**
GCS	0.909	0.882–0.936	**<0.001**	0.923	0.902–0.945	**<0.001**
Sofa score	1.128	1.1–1.156	**<0.001**	1.115	1.094–1.137	**<0.001**
SAPS II	1.048	1.042–1.054	**<0.001**	1.043	1.039–1.048	**<0.001**
APS III	1.028	1.024–1.032	**<0.001**	1.025	1.023–1.028	**<0.001**
OASIS	1.086	1.075–1.097	**<0.001**	1.070	1.062–1.078	**<0.001**
SIRS	1.621	1.467–1.791	**<0.001**	1.382	1.285–1.486	**<0.001**
Hypertension	1.234	0.943–1.613	0.125	0.960	0.799–1.154	0.666
Diabetes	0.883	0.726–1.075	0.217	1.029	0.892–1.188	0.694
Acute Myocardial infarct	1.144	0.9–1.453	0.272	1.131	0.945–1.353	0.180
Heart failure	1.331	1.091–1.623	**0.005**	1.489	1.287–1.723	**<0.001**
Peripheral vascular disease	0.800	0.602–1.063	0.123	0.921	0.753–1.126	0.421
Chronic pulmonary disease	1.018	0.81–1.279	0.877	1.196	1.016–1.408	**0.032**
Respiratory failure	1.822	1.495–2.22	**<0.001**	1.620	1.392–1.886	**<0.001**
VAP	1.230	0.834–1.815	0.297	1.514	1.156–1.982	**0.003**
CKD	1.194	0.962–1.483	0.108	1.368	1.17–1.601	**<0.001**
Renal failure	2.239	1.783–2.81	**<0.001**	2.027	1.697–2.42	**<0.001**
Hyperlipidemia	1.014	0.843–1.221	0.879	0.982	0.856–1.128	0.800
Malignancy	2.026	1.55–2.65	**<0.001**	2.314	1.905–2.812	**<0.001**
Liver disease	0.925	0.664–1.290	0.647	1.186	0.946–1.486	0.140
Sepsis: = 1	1.698	0.521–5.527	0.380	0.968	0.54–1.735	0.912
2	3.555	1.132–11.165	**0.030**	1.676	0.961–2.925	0.069
3	5.582	1.784–17.469	**0.003**	2.232	1.282–3.889	**0.005**
4	9.205	2.917–29.041	**<0.001**	3.067	1.735–5.42	**<0.001**
CCI	1.224	1.186–1.263	**<0.001**	1.220	1.192–1.249	**<0.001**
Vasopressors	2.802	2.071–3.791	**<0.001**	2.247	1.747–2.891	**<0.001**
Oxygen	0.585	0.484–0.708	**<0.001**	0.822	0.709–0.954	**0.010**
CRRT	1.773	1.144–2.748	**0.010**	1.603	1.132–2.272	**0.008**
Thrombolysis	0.705	0.376–1.319	0.273	1.121	0.769–1.634	0.554
Thrombectomy	0.973	0.706–1.342	0.869	0.904	0.706–1.157	0.423
RBC	0.816	0.719–0.926	**0.002**	0.697	0.634–0.767	**<0.001**
WBC	1.043	1.031–1.055	**<0.001**	1.032	1.022–1.043	**<0.001**
Platelet	0.992	0.991–1.000	**0.023**	0.999	0.997–1.001	**0.026**
Hb	0.934	0.896–0.975	**0.002**	0.882	0.855–0.911	**<0.001**
Sodium	1.074	1.055–1.093	**<0.001**	1.048	1.032–1.063	**<0.001**
Potassium	0.885	0.751–1.044	0.147	0.926	0.82–1.046	0.215
BUN	1.012	1.008–1.016	**<0.001**	1.013	1.01–1.016	**<0.001**
Creatinine	1.062	1.000–1.129	0.051	1.063	1.016–1.112	**0.008**
Albumin	0.497	0.428–0.577	**<0.001**	0.524	0.468–0.586	**<0.001**
ALT	1.002	1.000–1.004	0.134	1.000	1.000–1.003	0.097
AST	1.001	1.000–1.002	0.081	1.000	1.000–1.002	**0.025**
LDH	1.001	1.000–1.002	**0.044**	1.000	1.000–1.001	**0.009**
TC	0.996	0.994–0.998	**<0.001**	0.996	0.995–0.998	**<0.001**
TG	0.998	0.997–0.999	**<0.001**	0.999	0.998–1	**0.005**
HDL	0.994	0.989–1.000	**0.032**	0.994	0.991–0.998	**0.004**
LDL	0.996	0.994–0.998	**<0.001**	0.996	0.994–0.998	**<0.001**
Anion gap	1.089	1.06–1.119	**<0.001**	1.062	1.039–1.084	**<0.001**
Lactate	1.067	1.038–1.098	**<0.001**	1.069	1.046–1.092	**<0.001**
PT	0.998	0.985–1.011	0.713	1.007	1.000–1.015	0.051
APTT	0.996	0.991–1.001	0.096	1.000	0.996–1.003	0.880
INR	1.015	0.892–1.154	0.823	1.116	1.031–1.208	**0.006**
TG/HDL	1.001	0.983–1.019	0.953	1.004	0.991–1.017	0.577
TC/HDL	0.988	0.935–1.044	0.664	0.992	0.952–1.034	0.707
LDL/HDL	0.979	0.903–1.062	0.613	0.959	0.900–1.021	0.189
WBC/HDL	2.458	1.921–3.145	**<0.001**	2.141	1.711–2.679	**<0.001**

Bold values (*P* < 0.05) indicate that the corresponding variables are significantly associated with all-cause mortality in the Cox regression analysis.

Following the assessment of multicollinearity (Supplementary Tables 3, 4), six multivariable Cox regression models were constructed, adjusting for covariates such as age, gender, SOFA score, and other clinically relevant factors (Table 4). Each model demonstrated minimal multicollinearity, indicated by low variance inflation factors (VIFs). Across all models, the WBC/HDL ratio remained a statistically significant independent predictor for both mortality endpoints. Adjusted HRs for 28-day mortality ranged from 2.198 to 3.225, and for 1-year mortality from 1.864 to 3.163 (all *p* < 0.001). These results underscore the robustness of the WBC/HDL ratio as a mortality predictor, even after controlling for potential confounding variables.

**Table 4:** Multivariate Cox regression analysis of lipid profiles and their ratios associated with 28-day and 1-year all-cause mortality in patients with ischemic stroke.

SpO_2_	SpO_2_	SpO_2_			SpO_2_		
		SpO_2_	SpO_2_	*p*	HR	95%CI	*p*
Model 1	TG	0.998	0.997–0.999	**<0.001**	0.999	0.998–1	**<0.001**
	HDL	1.000	0.994–1.006	0.994	0.999	0.995–1.003	0.659
	WBC/HDL	2.947	2.203–3.942	**<0.001**	3.163	1.945–3.334	**<0.001**
Model 2	Age	1.042	1.034–1.051	**<0.001**	1.042	1.036–1.048	**<0.001**
	Gender	0.917	0.754–1.114	0.382	0.889	0.769–1.028	0.113
	TG	0.999	0.998–1	**0.021**	1.000	0.999–1	0.255
	HDL	0.997	0.991–1.003	0.295	0.995	0.991–1	**0.045**
	WBC/HDL	3.225	2.378–4.375	**<0.001**	2.757	2.088–3.64	**<0.001**
Model 3	Age	1.028	1.018–1.037	**<0.001**	1.027	1.02–1.034	**<0.001**
	Gender	0.900	0.741–1.093	0.286	0.877	0.759–1.014	0.076
	CCI	1.149	1.108–1.192	**<0.001**	1.159	1.127–1.191	**<0.001**
	TG	0.999	0.998–1	**0.025**	1.000	0.999–1	0.253
	HDL	0.998	0.992–1.004	0.560	0.997	0.992–1.001	0.139
	WBC/HDL	2.786	2.039–3.806	**<0.001**	2.341	1.764–3.108	**<0.001**
Model 4	Age	1.041	1.032–1.049	**<0.001**	1.041	1.035–1.047	**<0.001**
	Gender	0.853	0.702–1.036	0.109	0.827	0.715–0.956	0.010
	SOFA Score	1.127	1.096–1.158	**<0.001**	1.122	1.099–1.146	**<0.001**
	TG	0.998	0.997–0.999	**0.002**	0.999	0.999–1	**0.025**
	HDL	0.997	0.991–1.003	0.292	0.995	0.991–1	4.350
	WBC/HDL	2.505	1.842–3.406	**<0.001**	2.183	1.653–2.883	**<0.001**
Model 5	Age	1.030	1.021–1.04	**<0.001**	1.029	1.022–1.036	**<0.001**
	Gender	0.821	0.675–0.998	**0.047**	0.806	0.697–0.933	**0.004**
	CCI	1.129	1.087–1.173	**<0.001**	1.141	1.109–1.173	0.041
	SOFA Score	1.088	1.056–1.122	**<0.001**	1.091	1.066–1.116	**<0.001**
	Vasopressors	2.021	1.432–2.853	**<0.001**	1.761	1.33–2.33	**<0.001**
	TG	0.998	0.997–1	**0.004**	0.999	0.999–1	**0.041**
	HDL	0.998	0.992–1.004	0.452	0.996	0.992–1.001	0.098
	WBC/HDL	2.229	1.635–3.041	**<0.001**	1.931	1.46–2.555	**<0.001**
Model 6	Age	1.030	1.021–1.04	**<0.001**	1.029	1.022–1.037	**<0.001**
	Gender	0.817	0.672–0.995	**0.044**	0.808	0.698–0.934	**0.004**
	CCI	1.129	1.088–1.173	**<0.001**	1.141	1.109–1.174	**<0.001**
	SOFA Score	1.084	1.05–1.118	**<0.001**	1.082	1.057–1.108	**<0.001**
	Vasopressors	1.940	1.342–2.803	**<0.001**	1.561	1.16–2.101	**0.003**
	Platelet	1.000	0.999–1.001	0.598	1.000	0.999–1.001	0.797
	Lactate	1.014	0.979–1.051	0.431	1.038	1.012–1.066	**0.005**
	TG	0.998	0.997–1	**0.004**	0.999	0.999–1	**0.029**
	HDL	0.998	0.992–1.003	0.427	0.996	0.991–1	0.065
	WBC/HDL	2.198	1.613–2.995	**<0.001**	1.864	1.409–2.466	**<0.001**

Bold values (*P* < 0.05) indicate that the corresponding variables are significantly associated with all-cause mortality in the Cox regression analysis.

**Figure 2:**
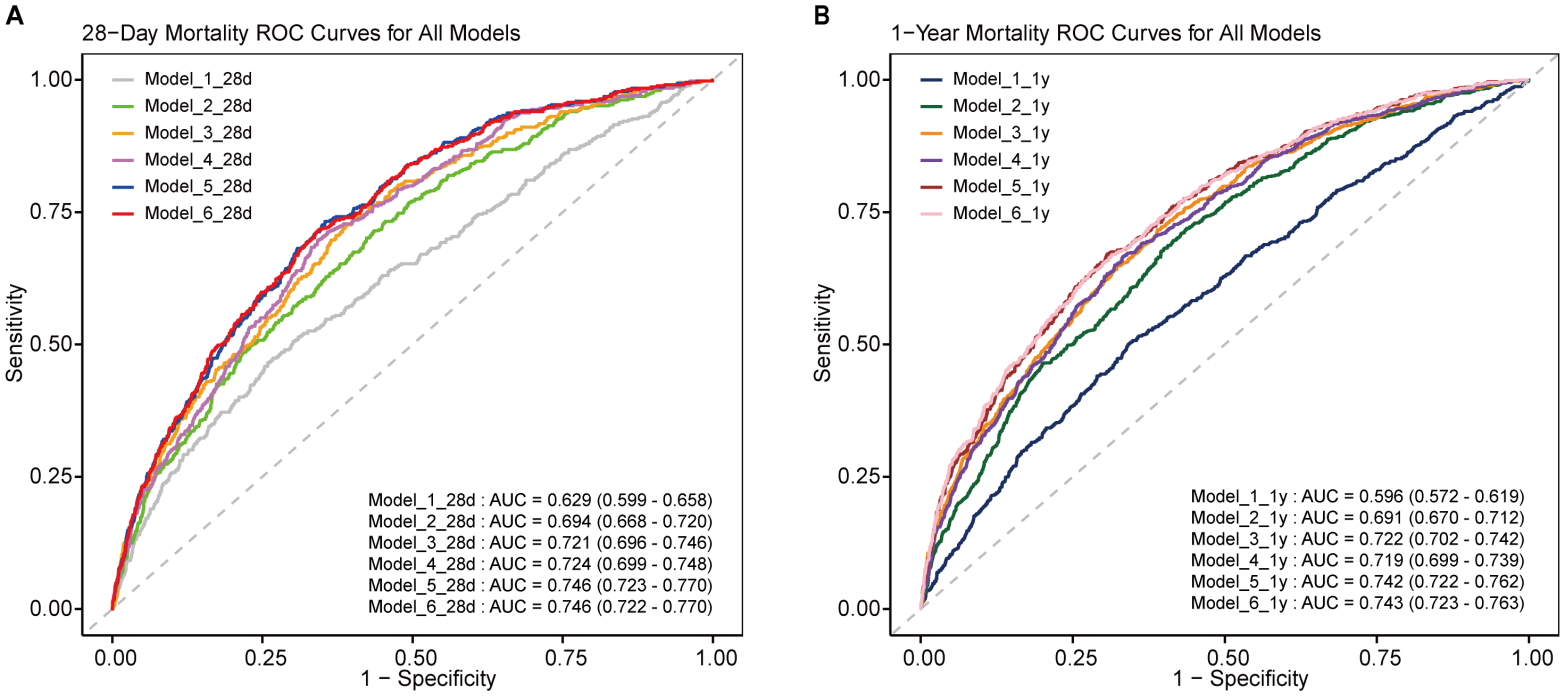
ROC curves of multiple models for predicting 28-day and 1-year mortality. ROC curves for all multiple models, showing the predictive performance for 28-day and 1-year mortality in ICU-admitted ischemic stroke patients. **(A)** 28-Day mortality ROC curve for all models; **(B)** 1-year mortality ROC curve for all models. AUC values and 95% confidence intervals are included for each curve.

To evaluate the predictive performance of the multivariable models, ROC curves were analyzed (Figure 2). Models incorporating the WBC/HDL ratio consistently achieved higher AUCs, highlighting its prognostic value for mortality risk stratification. Specifically, for Model 6 for 28-day mortality, the model incorporating the WBC/HDL ratio achieved an AUC of 0.746 (95% CI: 0.722–0.770), a sensitivity of 71.7%, and a specificity of 34.4%. In contrast, the AUC for Model 6 without WBC/HDL was 0.737 (95% CI: 0.713–0.761), with a sensitivity of 69.5% and a specificity of 33.2%. Similarly, for 1-year mortality, the AUC, sensitivity, and specificity of Model 6 incorporating WBC/HDL were higher than those of the model without WBC/HDL. The AUC for Model 6 incorporating WBC/HDL was 0.744, with a sensitivity of 64.6% and a specificity of 27.8%. In contrast, the AUC for Model 6 without WBC/HDL was 0.741, with a sensitivity of 61.5% and a specificity of 25.0%. These findings suggest that incorporating the WBC/HDL ratio into mortality prediction models improves predictive power, outperforming traditional lipid measurements, and support its clinical utility in risk stratification of patients with ischemic stroke.

### 3.3 RCS and Kaplan–Meier survival analyses

**Figure 3:**
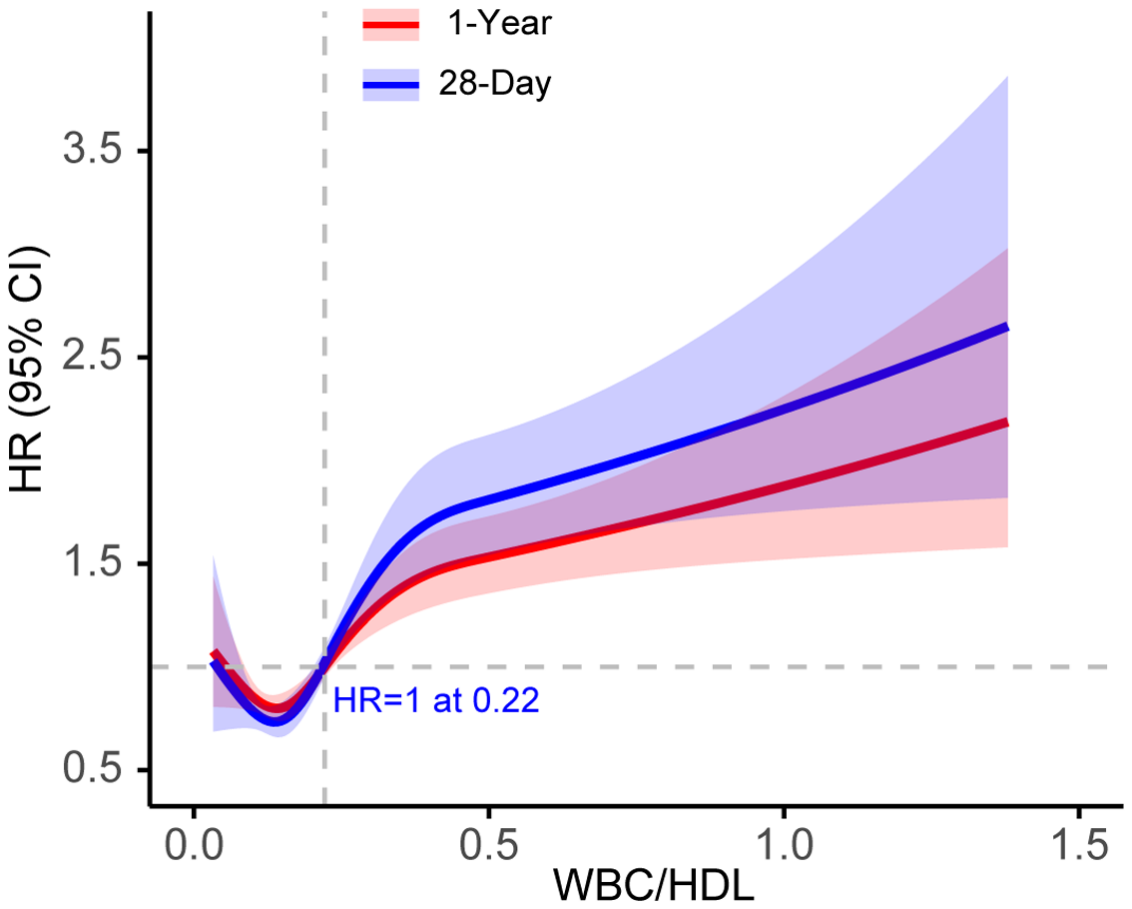
RCS curves for 28-day and 1-year mortality based on WBC/HDL ratio in ischemic stroke patients. RCS curve illustrating the association between WBC/HDL ratio and mortality risk at 28-day (Blue) and 1-year (Red) endpoints. The solid line represents the hazard ratio (HR), and shaded areas indicate 95% confidence intervals. The dashed horizontal line at HR = 1 indicates the reference risk level.

Using Youden’s index, we determined the optimal cutoff value (0.2828) to stratify patients into high and low WBC/HDL ratio groups. Figure 3 illustrates the RCS analysis for the WBC/HDL ratio, revealing a significant nonlinear relationship with both 28-day and 1-year mortality. The curve demonstrates a marked increase in mortality risk associated with higher WBC/HDL ratios, with a notable inflection point near the HR of 1. This pattern indicates that elevated WBC/HDL ratios are strongly correlated with increased mortality risk, supporting its role as a prognostic marker in patients with IS.

Supplementary Figure 1 presents the RCS curves for TG and HDL. Panel A displays the RCS curve for TG, and Panel B shows the curve for HDL. Both TG and HDL curves exhibit relatively flat slopes near the HR = 1 line, suggesting a weaker association with mortality risk compared to the WBC/HDL ratio. These findings imply that TG and HDL-C levels alone may have limited predictive value for mortality in this patient population.

**Figure 4:**
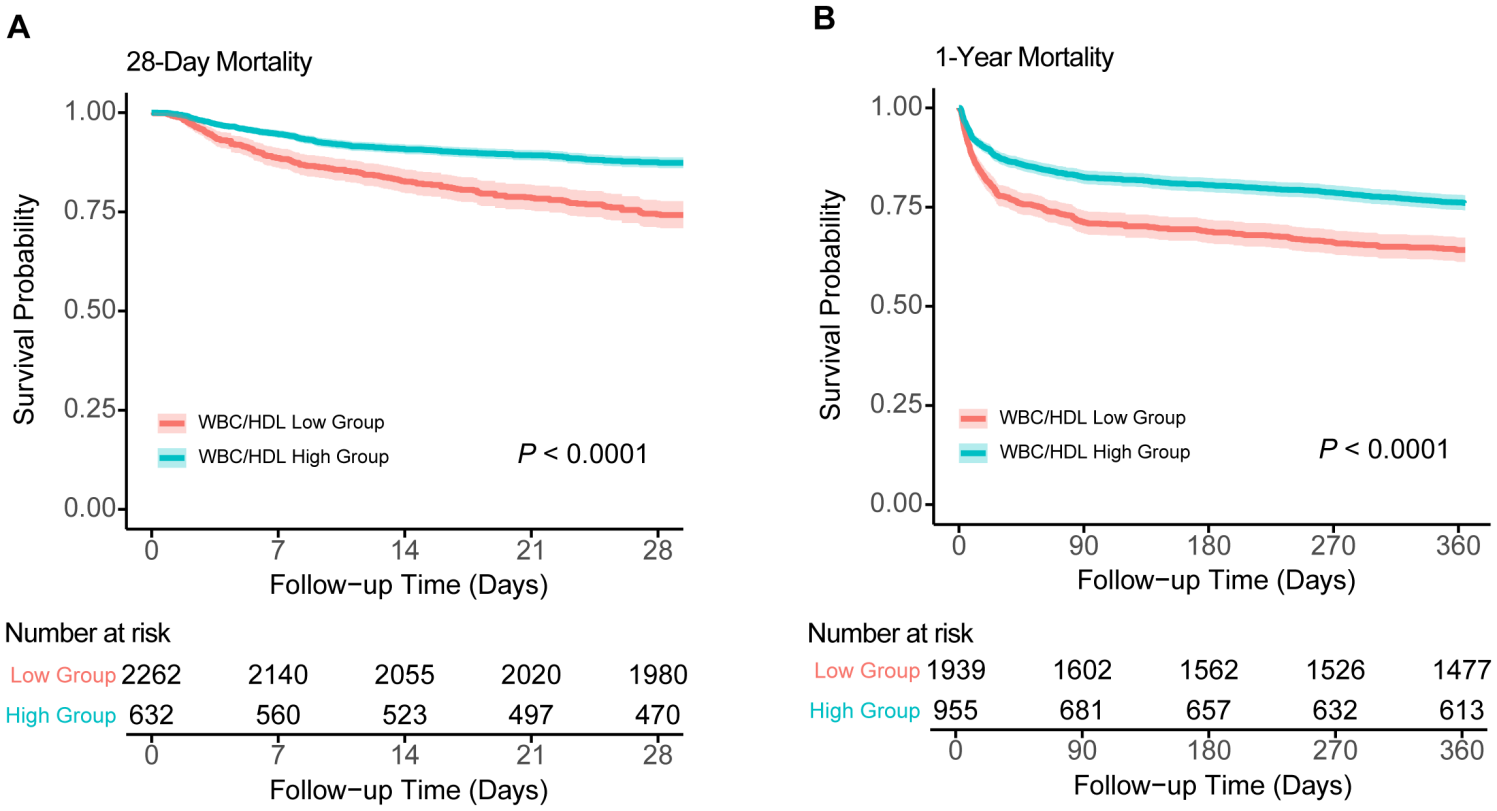
Kaplan–Meier survival analysis curves for 28-day and 1-year all-cause mortality. Kaplan–Meier survival curves comparing survival probabilities between patients stratified by WBC/HDL ratio into low and high groups, with number at risk shown at various time points. **(A)** Survival probabilities for 28-day mortality. **(B)** Survival probabilities for 1-year mortality. Log-rank test *p*-values indicate statistical significance.

Kaplan–Meier survival analyses further reinforce the prognostic significance of the WBC/HDL ratio (Figure 4). Patients with higher WBC/HDL ratios had significantly lower survival rates at both 28 days and 1 year compared to those with lower ratios (*p* < 0.001, log-rank test). The distinct separation of survival curves between the high and low WBC/HDL groups underscores the utility of this ratio as a predictor of both short-term and long-term mortality. In contrast, Kaplan–Meier curves for TG and HDL groups showed more modest or negligible differences in survival, aligning with the RCS findings and highlighting the superior predictive value of the WBC/HDL ratio over traditional lipid measures.

### 3.4 Subgroup analysis and forest plots

**Figure 5:**
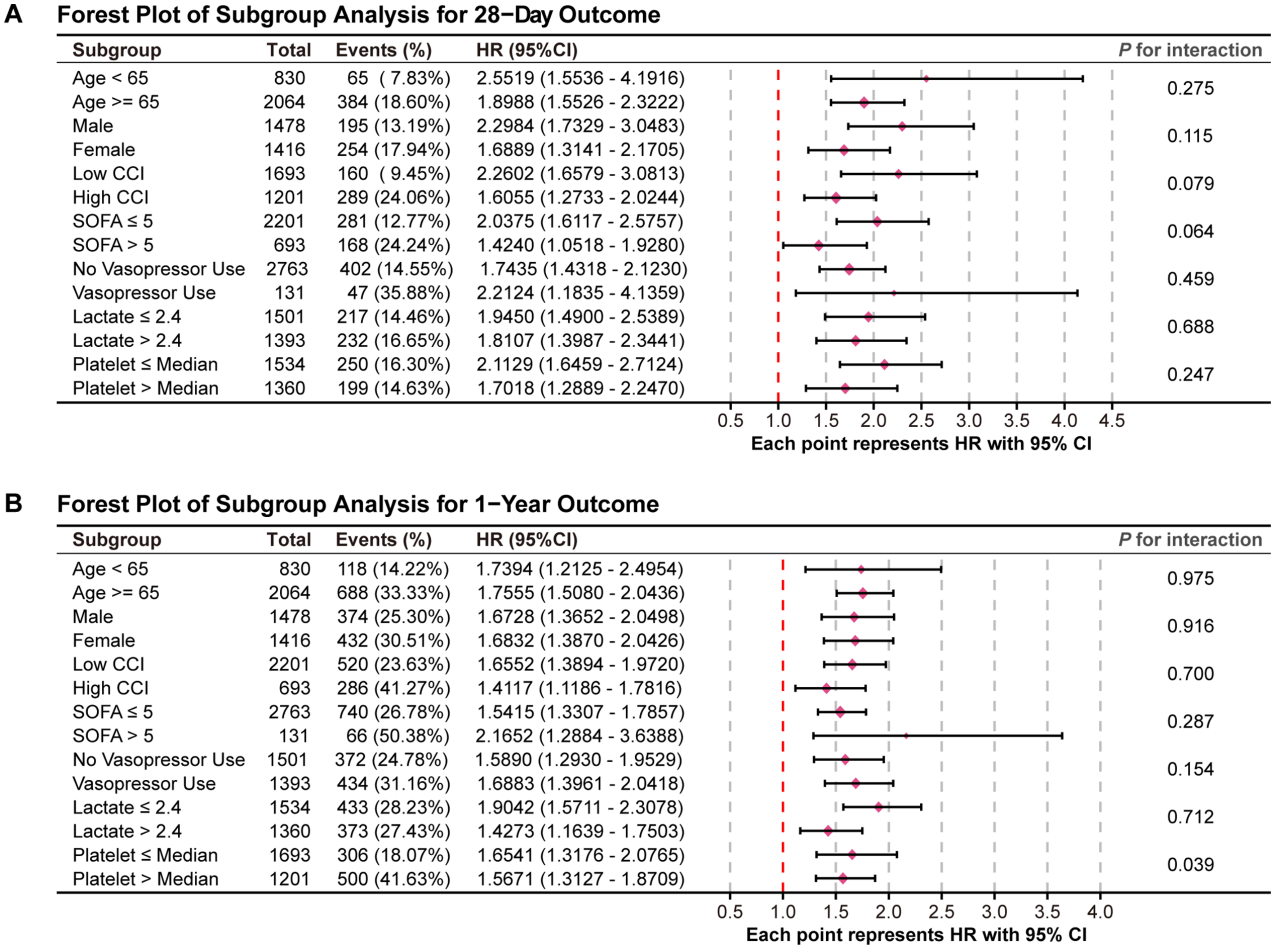
Forest plots for the subgroup analysis of the relationship between hospital mortality and WBC/HDL ratio. Forest plots summarizing the subgroup analysis of the association between WBC/HDL ratio and mortality risk. **(A)** Forest plot for the 28-day mortality outcome; **(B)** Forest plot for the 1-year mortality outcome. Subgroups include age, gender, and other relevant factors, with interaction *p*-values provided to assess the consistency of the WBC/HDL ratio effect across each subgroup.

Figure 5 displays the results of the subgroup analyses and corresponding forest plots examining the association between the WBC/HDL ratio and all-cause mortality at 28 days and 1 year following ICU admission in patients with ischemic stroke (Figure 5). Subgroups were stratified based on clinically relevant factors, including age, gender, CCI, SOFA score, vasopressor use, lactate levels, and platelet count.

In the 28-day mortality analysis (Figure 5A), the association between an elevated WBC/HDL ratio and increased mortality risk remained consistent across all subgroups, with no significant interaction effects observed (all *P* for interaction > 0.05). This indicates that the prognostic impact of the WBC/HDL ratio on 28-day mortality is stable and independent of these demographic and clinical characteristics.

For the 1-year mortality outcome (Figure 5B), the WBC/HDL ratio continued to be strongly associated with increased mortality risk across the various subgroups analyzed. Notably, a significant interaction effect was observed with platelet count (*P* for interaction = 0.039), suggesting that the relationship between the WBC/HDL ratio and 1-year mortality risk may vary depending on platelet levels. Specifically, the prognostic value of the WBC/HDL ratio appeared more pronounced in patients with lower platelet counts. This finding highlights a potential modifying effect of platelet count on the long-term prognostic significance of the WBC/HDL ratio.

In addition to the subgroup analyses based on clinical and laboratory factors, we further explored potential interactions between the WBC/HDL ratio and specific patient characteristics, such as hypertension, diabetes, heart failure, CRRT, and thrombolysis/thrombectomy treatment. The interaction analysis revealed no significant interactions between WBC/HDL ratio and hypertension (*P* of interaction = 0.941 [28-day] and 0.499 [1-year]), heart failure (*P* of interaction = 0.835 [28-day] and 0.533 [1-year]), or CRRT (*P* of interaction = 0.492 [28-day] and 0.867 [1-year]). However, a borderline interaction effect was observed with diabetes (*p* = 0.0605 [1-year]), suggesting a potential modifying effect of diabetes on the prognostic value of WBC/HDL ratio in the 1-year endpoint. Similarly, no significant interaction was found for thrombolysis/thrombectomy (*P* of interaction = 0.96 [28-day] and 0.661 [1-year]). These findings underscore the robustness of WBC/HDL as a prognostic marker in ischemic stroke patients, with potential variations in predictive value across subgroups. The results of this interaction analysis are presented in Supplementary Figure 2.

Overall, these results underscore the robustness of the WBC/HDL ratio as an independent predictor of both short-term and long-term mortality in ischemic stroke patients, largely unaffected by most demographic and clinical factors examined. However, the observed interaction with platelet count in the 1-year analysis suggests that further investigation is warranted to understand the underlying mechanisms and to determine how platelet levels may influence the prognostic utility of the WBC/HDL ratio in long-term risk stratification.

## 4 Discussion

It is well known that dyslipidemia contributes to cardiovascular and cerebrovascular atherosclerotic diseases. In our retrospective cohort study using data from the MIMIC-IV database, we aimed to assess the prognostic significance of lipid profiles and their relative ratios in patients with ischemic stroke. I Initially, we hypothesized that traditional lipid parameters—including TC, TG, LDL, and HDL—and their respective ratios (LDL/HDL, TC/HDL, TG/HDL) would be associated with mortality risk in critically ill ischemic stroke patients. In our study, these traditional lipid measures were indeed significantly different between survivors and non-survivors in baseline comparisons and showed significance in univariate Cox regression analyses. This suggests that, at a univariate level, there is an association between lipid levels and mortality outcomes.

However, when we evaluated their predictive performance using ROC curve analyses, the AUC values for these lipid parameters were relatively low, indicating limited discriminative ability. Furthermore, in multivariate Cox regression models adjusting for potential confounders, as well as in RCS analyses, these traditional lipid parameters did not retain statistical significance. This attenuation of significance suggests that their initial associations with mortality may be confounded by other clinical factors or that they do not independently predict mortality risk when considered alongside other variables.

The lack of consistent predictive value of traditional lipid profiles in our study could be attributed to the complex metabolic disturbances induced by acute ischemic stroke and critical illness. Previous studies have documented that acute stroke activates a cascade of inflammatory and stress responses that substantially disrupt lipid metabolism (15–17). This includes reduced cholesterol synthesis and lipid particle redistribution driven by heightened catabolism and oxidative stress. These acute-phase reactions may diminish the prognostic value of traditional lipid markers in critically ill patients, as lipid levels may not accurately reflect baseline cardiovascular risk in this context (18, 19). Such metabolic responses could especially impact cholesterol and triglyceride levels, thereby limiting their utility as standalone prognostic indicators in critically ill patients (20, 21). Moreover, the phenomenon known as the “cholesterol paradox” may partially explain our findings. In critically ill patients, lower cholesterol levels have paradoxically been associated with increased mortality (22, 23). Hypocholesterolemia in this context may indicate a systemic inflammatory response or malnutrition, both of which are linked to poorer outcomes (19, 24). Acute-phase reactions can reduce lipid levels independently of baseline cholesterol status, further diminishing the utility of traditional lipid measurements for prognostication in acute settings (15, 25).

Given these limitations of traditional lipid parameters, alternative markers that remain reliable in the face of metabolic alterations are needed. In contrast to the traditional lipid profiles, the WBC/HDL ratio emerged as a significant independent predictor of mortality in our study. This ratio integrates both inflammatory status (via white blood cell count) and lipid metabolism (via HDL levels), providing a more comprehensive and stable indicator of the patient’s physiological state during critical illness. Elevated WBC reflects systemic inflammatory responses implicated in endothelial dysfunction, plaque instability, and secondary neuronal injury after stroke (26–28). This inflammatory response, driven by elevated WBC counts, contributes significantly to the exacerbation of ischemic injury. High WBC levels can disrupt the blood–brain barrier, leading to increased cerebral edema and further neuronal damage (29). The inflammatory milieu created by elevated WBC counts can also promote thrombus formation and worsen thromboembolism, compounding the ischemic insult. Furthermore, high WBC levels are associated with a heightened risk of recurrent ischemic events, as well as poor long-term neurological outcomes, due to the continued inflammatory response and microvascular damage (30). In ischemic stroke patients, this systemic inflammation not only accelerates the primary damage but also facilitates secondary injury, including excitotoxicity, oxidative stress, and apoptosis, all of which contribute to worse clinical outcomes.

HDL, recognized for its anti-inflammatory and antioxidative properties, plays a critical role in promoting endothelial repair and inhibiting LDL oxidation, thus providing protective effects against the development of atherosclerosis (31–33). These protective functions of HDL are particularly important in reducing the risk of atherosclerosis and ischemic stroke. In fact, HDL’s ability to modulate inflammation may be even more crucial than its concentration, further supporting HDL’s protective role in cardiovascular health (34). In contrast, a high WBC/HDL ratio indicates a state of elevated systemic inflammation, coupled with a reduction in protective lipid factors. This imbalance may exacerbate the progression of atherosclerosis and ischemic stroke, contributing to poorer patient outcomes. Therefore, the WBC/HDL ratio serves as a valuable indicator of the interplay between lipid metabolism and inflammation, both of which are pivotal in stroke pathophysiology.

Our findings align with a growing body of evidence suggesting that combined markers of inflammation and lipid metabolism provide greater prognostic value than traditional lipid profiles alone. Previous studies, including our own analysis on ICU-admitted cerebrovascular patients, have shown that inflammatory markers such as high-sensitivity C-reactive protein (hs-CRP) and the neutrophil-to-lymphocyte ratio (NLR) are associated with adverse outcomes in stroke patients (35–37). Ratios like the monocyte-to-HDL ratio have also been identified as predictors of mortality in cardiovascular diseases, including ischemic stroke (38–40). For instance, Wang et al. and Sun et al. both found that a higher monocyte-to-HDL ratio was independently associated with increased mortality risk in acute ischemic stroke patients (39, 40). Furthermore, not only monocytes but also neutrophils, as indicated by their ratio to high-density lipoprotein (NHR), have been shown to predict adverse stroke outcomes. Recent studies underscore NHR’s role in predicting hemorrhagic transformation in acute ischemic stroke, with higher NHR levels linked to an elevated risk of bleeding (41). Additionally, elevated NHR has been positively correlated with stroke severity, reflected in NIH Stroke Scale (NIHSS) scores, and identified as an independent risk factor for ischemic stroke, highlighting this ratio’s potential as a valuable prognostic tool in acute stroke management (42). These findings reinforce the hypothesis that combining inflammatory and lipid markers can improve risk stratification in stroke patients, offering a more comprehensive approach to assessing prognosis.

In the context of ischemic stroke, where acute management is paramount, clinical scales like NIHSS and imaging-based markers like infarct volume have long been established as key predictors of stroke severity and mortality (43, 44). While these markers provide valuable information, they focus primarily on the immediate neurological severity and infarct size, but they do not address the inflammatory and metabolic pathways that may significantly influence patient outcomes, especially in the ICU setting. Our study specifically focuses on the WBC/HDL ratio, a marker that integrates both inflammation and lipid metabolism. The WBC/HDL ratio offers complementary prognostic information beyond what is provided by traditional clinical scales and imaging markers, making it a promising tool for early risk stratification in critically ill stroke patients.

Selecting the most accessible and practical marker for clinical use is crucial. Among various leukocyte ratios (e.g., monocyte, neutrophil, and lymphocyte to HDL ratios), the WBC/HDL ratio stands out as an efficient, cost-effective tool that leverages routine laboratory tests. Our study demonstrated a robust association between elevated WBC/HDL ratios and increased 28-day and 1-year mortality, even after adjusting for multiple confounders. This highlights its potential utility as an effective prognostic marker for risk stratification in ICU-admitted ischemic stroke patients. Unlike traditional clinical scales such as NIHSS, which mainly assess neurological severity, or inflammatory biomarkers like CRP, the WBC/HDL ratio integrates both inflammatory and lipid metabolic pathways, offering complementary information that can enhance stroke prognosis prediction. Early identification of high-risk patients using this ratio can facilitate targeted interventions, including intensified monitoring, optimized medical therapy, and potentially anti-inflammatory treatments. Furthermore, the identification of elevated WBC/HDL ratios may prompt further investigation into systemic inflammation, modifiable risk factors, and the interaction between lipid metabolism and inflammation, ultimately improving patient care.

Building upon the significance of the WBC/HDL ratio observed in our analyses, we developed multivariate Cox proportional hazards models to evaluate its independent prognostic value for mortality in critically ill ischemic stroke patients. These models consistently demonstrated that an elevated WBC/HDL ratio was a significant predictor of both 28-day and 1-year all-cause mortality. After adjusting for potential confounders—including age, gender, comorbidities, and clinical severity scores—the WBC/HDL ratio remained an independent risk factor. Specifically, patients in the high WBC/HDL ratio group had nearly a twofold higher risk of 28-day mortality (adjusted hazard ratio [HR]: 2.198; 95% confidence interval [CI]: 1.864–3.225; *p* < 0.001) and more than a twofold higher risk of 1-year mortality (adjusted HR: 3.163; 95% CI: 2.947–3.334; *p* < 0.001) compared to those in the low ratio group. These findings underscore the robustness of the WBC/HDL ratio as a prognostic marker, independent of other established risk factors.

The enhanced predictive power of our models was further supported by ROC curve analyses. Inclusion of the WBC/HDL ratio significantly improved the AUC, indicating better discriminative ability for mortality outcomes. For instance, the AUC for 28-day mortality prediction increased from 0.621 to 0.686 upon adding the WBC/HDL ratio to the model, surpassing the predictive performance of models utilizing traditional lipid parameters alone. This improvement highlights the added value of incorporating the WBC/HDL ratio into prognostic models for ischemic stroke patients in the ICU.

Moreover, Kaplan–Meier survival analysis provided clear evidence of the effect of the WBC/HDL ratio on patient survival. Patients with an elevated WBC/HDL ratio had a significantly lower probability of survival at 28 days and 1 year after admission compared with those with a lower WBC/HDL ratio (log-rank *p* < 0.001). As shown in the figures of our study, the K-M survival curves diverged early and continued to separate over time, indicating that the ratio was closely associated with the risk of death. These results not only validate the prognostic importance of the WBC/HDL ratio but also demonstrate its potential utility in stratifying patients according to the risk of death in clinical practice.

Our subgroup analyses reinforced the consistency of the WBC/HDL ratio’s predictive value across various patient demographics and clinical conditions. The ratio remained a significant predictor of mortality regardless of age, gender, comorbidity burden, or severity of illness scores. Notably, an interaction effect was observed with platelet count in the 1-year mortality analysis (*P* for interaction = 0.039), suggesting that the prognostic impact of the WBC/HDL ratio may be modulated by platelet levels. This finding aligns with existing literature indicating that platelet function and counts can influence inflammatory processes and thrombosis, thereby affecting stroke outcomes (45, 46). Thrombocytopenia may indicate increased platelet consumption due to ongoing microthrombosis or bone marrow suppression from systemic inflammation (45, 47, 48). Further research is needed to explore this interaction and its clinical implications. Our findings resonate with Li et al.’s demonstration of residual inflammatory risk (RIR, defined by hsCRP ≥3 mg/L with controlled LDL-C) as a key predictor of poor stroke outcomes (49). While their work highlighted inflammation’s primacy—showing RIR independently predicted 1-year stroke recurrence (adjusted HR 1.18) and functional disability (adjusted OR 1.43)—our study advances this paradigm by revealing that combining inflammatory (WBC) and lipid (HDL) markers provides superior prognostic value. Specifically, the WBC/HDL ratio captures both: (1) the inflammatory burden emphasized by Li et al., and (2) HDL’s protective effects against neuroinflammation—a dimension not assessed in their hsCRP/LDL-C framework. Notably, Li et al. found RIR’s impact was strongest in large-artery atherosclerosis (LAA) patients (HR 1.69 with LDL-C < 1.8 mmol/L), suggesting our observed WBC/HDL predictive power may be particularly relevant for LAA subtypes, warranting future subtype-specific validation. While NIHSS and infarct volume remain important markers for assessing stroke severity, the WBC/HDL ratio may offer added prognostic value when combined with these clinical scales in predicting long-term outcomes in stroke patients. We suggest that future studies explore how the WBC/HDL ratio interacts with established markers such as NIHSS and infarct volume to improve overall risk stratification and guide more personalized treatment strategies. Additionally, subgroup analyses based on NIHSS severity score and infarct volume could further illuminate how the WBC/HDL ratio behaves within different patient populations and clinical settings.

Despite the strengths of our study, several limitations should be acknowledged. First, as a retrospective analysis, it is subject to potential selection bias and residual confounding, despite adjustments for known variables. Second, the data were derived from a single-center database, which may limit the generalizability of our findings to other populations and healthcare settings. Therefore, future studies should validate these findings in diverse settings, including different healthcare systems and ethnic/racial groups. Additionally, assessing the performance of the WBC/HDL ratio in ischemic stroke patients outside of the ICU could provide important insights into its applicability in broader patient populations.

Third, we did not account for interventions during hospitalization that could affect WBC and HDL levels—such as infections, medications (e.g., corticosteroids, statins), or nutritional support—which may influence the WBC/HDL ratio and potentially confound its association with mortality. Future studies should examine the dynamic changes in the WBC/HDL ratio over time and explore whether interventions targeting inflammation or lipid profiles can improve outcomes in patients with elevated ratios. While our study focused on the WBC/HDL ratio at ICU admission, its predictive value may change throughout the ICU stay and recovery. Investigating these changes at multiple time points could enhance the understanding of its role in ischemic stroke prognosis.

Investigating whether interventions aimed at reducing inflammation or modifying lipid profiles can improve outcomes in patients with elevated WBC/HDL ratios would also be of clinical interest. Additionally, exploring the mechanisms underlying the interaction between the WBC/HDL ratio and platelet count could offer insights into the pathophysiology of ischemic stroke and reveal potential therapeutic targets.

## 5 Conclusion

In conclusion, our study demonstrates that the WBC/HDL ratio is a significant independent predictor of both short-term (28-day) and long-term (1-year) mortality in critically ill patients with ischemic stroke, outperforming traditional lipid measures. By integrating inflammatory and lipid components, the ratio offers a more comprehensive marker of mortality risk. Incorporating the WBC/HDL ratio into clinical practice may enhance risk stratification and guide personalized management strategies, ultimately improving patient outcomes. Future prospective studies are warranted to validate these findings and explore interventions targeting the WBC/HDL ratio.

## Data Availability

The original contributions presented in the study are included in the article/Supplementary material, further inquiries can be directed to the corresponding authors.

## Footnotes

^1^ https://physionet.org/

## Supplementary material

SUPPLEMENTARY FIGURE 1 RCS curves for 28-day and 1-year mortality based on TG and HDL in ischemic stroke patients. **(A)** RCS curves for TG levels with 28-day (blue) and 1-year (red) mortality outcomes. Dashed lines show HR = 1 points: 117.39 mg/dL (28-day) and 133.95 mg/dL (1-year). **(B)** RCS curves for HDL levels with 28-day (blue) and 1-year (red) mortality outcomes. Dashed lines show HR=1 points: 37.95 mg/dL (28-day) and 36.64 mg/dL (1-year). Shaded areas indicate 95% confidence intervals.

## Glossary

APACHE III: Acute Physiology and Chronic Health Evaluation III
AUC: Area Under the Curve
BUN: Blood Urea Nitrogen
CCI: Charlson Comorbidity Index
CKD: Chronic Kidney Disease
CI: Confidence Interval
CRP: C-Reactive Protein
FBG: Fasting Blood Glucose
HR: Hazard Ratio
hs-CRP: High-Sensitivity C-Reactive Protein
ICU: Intensive Care Unit
INR: International Normalized Ratio
IQR: Interquartile Range
IS: Ischemic Stroke
K-M: Kaplan–Meier
LDH: Lactate Dehydrogenase
MIMIC-IV: Medical Information Mart for Intensive Care IV
OR: Odds Ratio
PT: Prothrombin Time
RCS: Restricted Cubic Spline
ROC: Receiver Operating Characteristic
SAP II: Simplified Acute Physiology Score II
SCI: Science Citation Index
SD: Standard Deviation
SOFA: Sequential Organ Failure Assessment
TC: Total Cholesterol
TG: Triglycerides
VIF: Variance Inflation Factor
WBC: White Blood Cell
