# Supplementary figures and images for: The WBC/HDL ratio outperforms other lipid profiles in predicting mortality among ischemic stroke patients: a retrospective cohort study using MIMIC-IV data
Source: Front Neurol. 2025 Apr 30;16:1534381. doi: 10.3389/fneur.2025.1534381 (PMC12074928; doi:10.3389/fneur.2025.1534381)

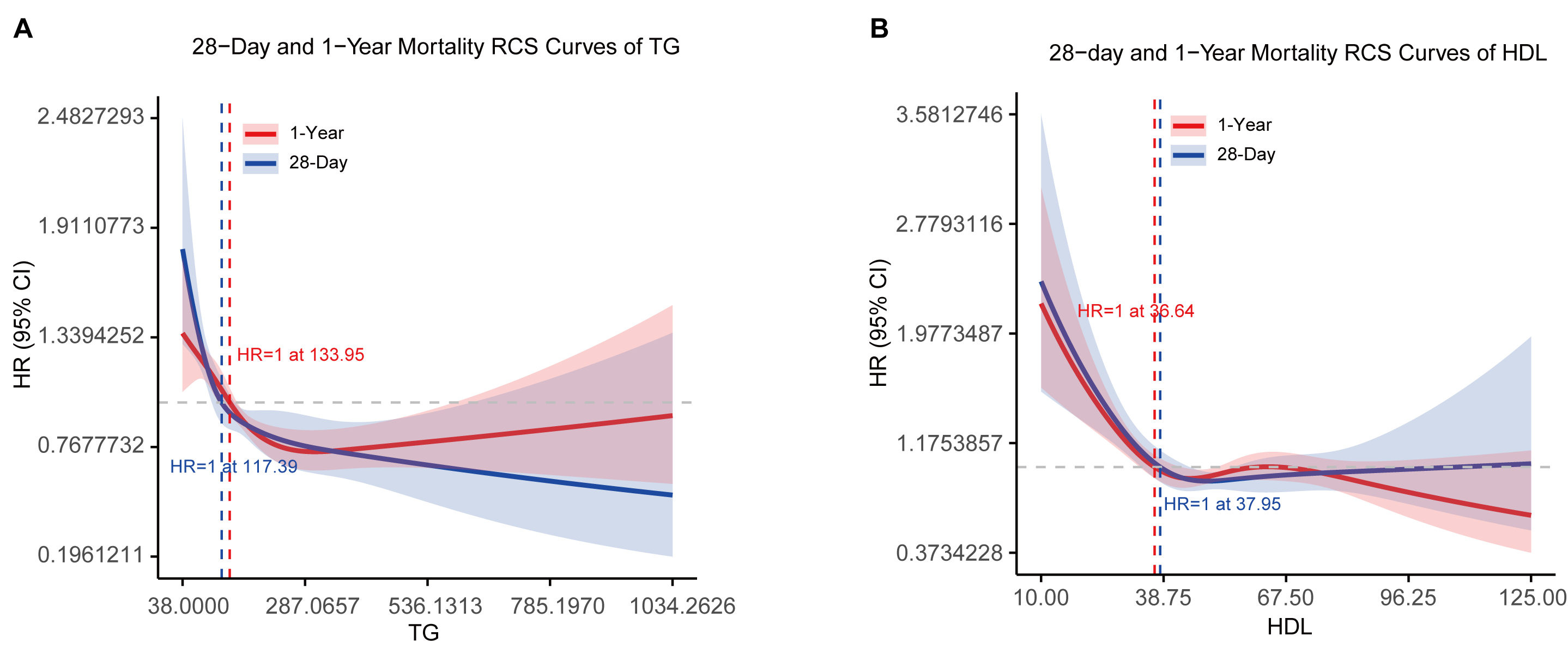

Supplement: SUPPLEMENTARY FIGURE 1 — RCS curves for 28-day and 1-year mortality based on TG and HDL in ischemic stroke patients. (A) RCS curves for TG levels with 28-day (blue) and 1-year (red) mortality outcomes. Dashed lines show HR = 1 points: 117.39 mg/dL (28-day) and 133.95 mg/dL (1-year). (B) RCS curves for HDL levels with 28-day (blue) and 1-year (red) mortality outcomes. Dashed lines show HR=1 points: 37.95 mg/dL (28-day) and 36.64 mg/dL (1-year). Shaded areas indicate 95% confidence intervals. [file Image_1.TIF]

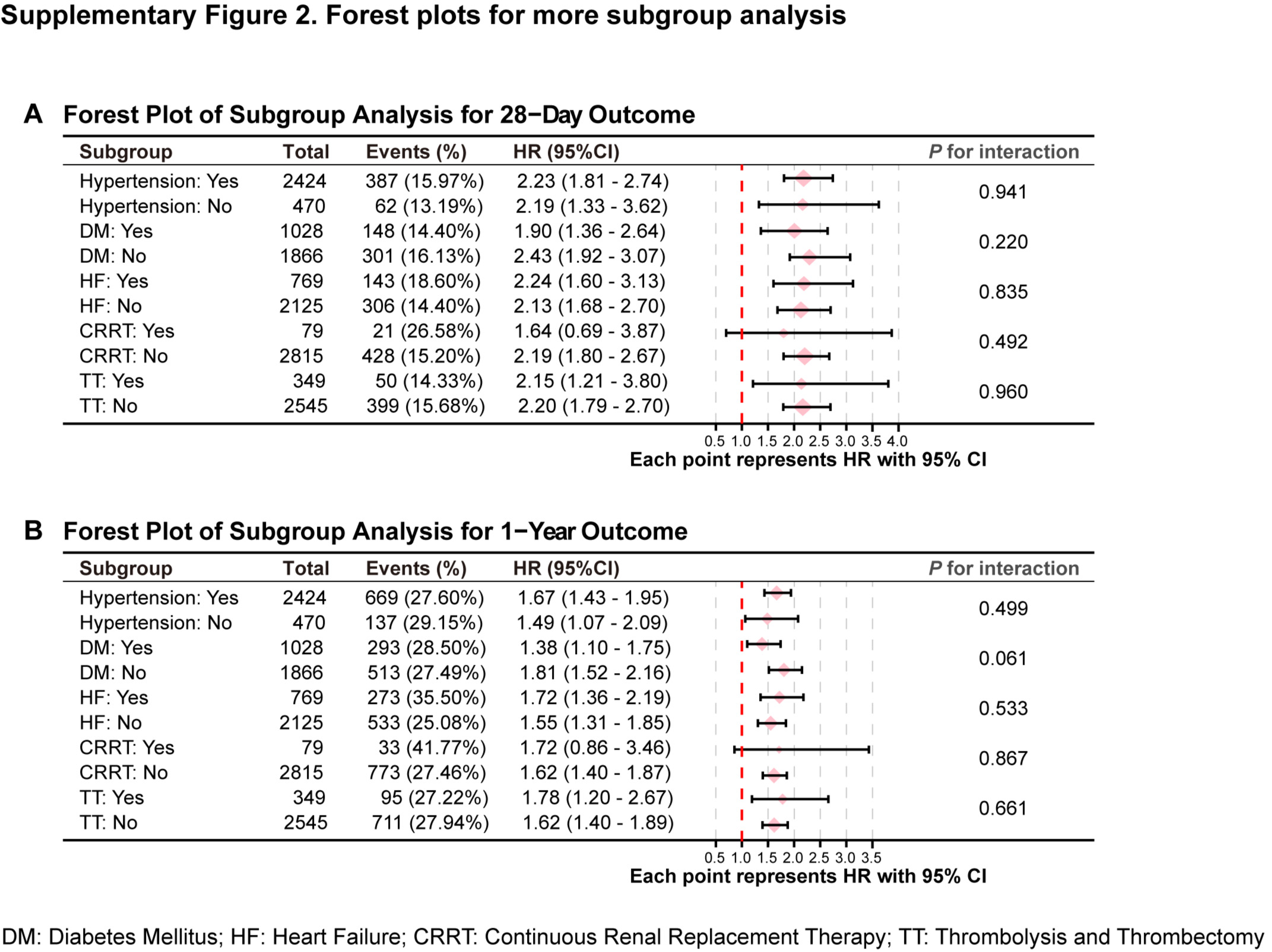

Supplement: Supplementary file 2 [file Image_2.JPEG]
