# Supplementary material for: The WBC/HDL ratio outperforms other lipid profiles in predicting mortality among ischemic stroke patients: a retrospective cohort study using MIMIC-IV data
Source: Front Neurol. 2025 Apr 30;16:1534381. doi: 10.3389/fneur.2025.1534381 (PMC12074928; doi:10.3389/fneur.2025.1534381)
Supplement: Supplementary file 3 [file Table_1.DOCX]

**Supplementary Table 1. Test of normality results of continuous variables**

| **Variable** | ***P* value*** | **Normality** |
| --- | --- | --- |
| Age | 1.41 🞨 10^-24^ | Not Normal |
| Height | 7.81 🞨 10^-18^ | Not Normal |
| Weight | 3.26 🞨 10^-29^ | Not Normal |
| MBP | 1.56 🞨 10^-17^ | Not Normal |
| Heart rate | 6.61 🞨 10^-18^ | Not Normal |
| SBP | 1.37 🞨 10^-13^ | Not Normal |
| DBP | 8.43 🞨 10^-16^ | Not Normal |
| Respirate rate | 1.03 🞨 10^-29^ | Not Normal |
| SpO2 | 1.72 🞨 10^-33^ | Not Normal |
| Glasgow coma score | 4.79 🞨 10^-66^ | Not Normal |
| SOFA score | 3.86 🞨 10^-40^ | Not Normal |
| SAPS II | 3.34 🞨 10^-26^ | Not Normal |
| APS III | 1.39 🞨 10^-35^ | Not Normal |
| OASIS | 4.20 🞨 10^-16^ | Not Normal |
| SIRS | 2.27 🞨 10^-39^ | Not Normal |
| RBC | 4.79 🞨 10^-06^ | Not Normal |
| Hb | 5.16 🞨 10^-08^ | Not Normal |
| Platelet | 1.04 🞨 10^-39^ | Not Normal |
| WBC | 1.89 🞨 10^-52^ | Not Normal |
| Sodium | 3.77 🞨 10^-32^ | Not Normal |
| Potassium | 1.25 🞨 10^-36^ | Not Normal |
| BUN | 1.48 🞨 10^-52^ | Not Normal |
| Creatinine | 4.71 🞨 10^-67^ | Not Normal |
| Albumin | 9.66 🞨 10^-24^ | Not Normal |
| ALT | 2.08 🞨 10^-75^ | Not Normal |
| AST | 2.73 🞨 10^-77^ | Not Normal |
| LDH | 3.51 🞨 10^-77^ | Not Normal |
| TC | 4.95 🞨 10^-28^ | Not Normal |
| TG | 2.27 🞨 10^-64^ | Not Normal |
| HDL | 3.91 🞨 10^-27^ | Not Normal |
| LDL | 3.58 🞨 10^-31^ | Not Normal |
| Anion gap | 1.07 🞨 10^-25^ | Not Normal |
| Lactate | 6.43 🞨 10^-59^ | Not Normal |
| PT | 1.17 🞨 10^-66^ | Not Normal |
| APTT | 4.82 🞨 10^-60^ | Not Normal |
| INR | 1.67 🞨 10^-65^ | Not Normal |
| TG/HDL | 9.93 🞨 10^-70^ | Not Normal |
| TC/HDL | 2.03 🞨 10^-54^ | Not Normal |
| LDL/HDL | 1.58 🞨 10^-47^ | Not Normal |
| WBC/HDL | 2.15 🞨 10^-62^ | Not Normal |

*: Kolmogorov-Smirnov Test

MBP: mean blood pressure; SBP: systolic blood pressure; DBP: diastolic blood pressure; SOFA score: Sequential Organ Failure Assessment Score; SAPS II: Simplified Acute Physiology Score II; APS III: Acute Physiology Score III; OASIS: Oxford Acute Severity of Illness Score; SIRS: Systemic Inflammatory Response Syndrome Score; RBC: red blood cell counts; WBC: white blood cell counts; Hb: Hemoglobin; BUN: blood urea nitrogen; ALT: Alanine Aminotransferases; AST: Aspartate Aminotransferase; LDH: Lactate Dehydrogenase; TC: triglyceride; TG: triglyceride; HDL: High-Density Lipoprotein; LDL: Low-Density Lipoprotein; PT: Prothrombin Time; APTT: Activated Partial Thromboplastin Time; INR: International Normalized Ratio; TG/HDL: TG to HDL ratio; TC/HDL: TC to HDL ratio; LDL/HDL: LDL to HDL ratio; WBC/HDL: WBC to HDL ratio
