# Supplementary material for: The WBC/HDL ratio outperforms other lipid profiles in predicting mortality among ischemic stroke patients: a retrospective cohort study using MIMIC-IV data
Source: Front Neurol. 2025 Apr 30;16:1534381. doi: 10.3389/fneur.2025.1534381 (PMC12074928; doi:10.3389/fneur.2025.1534381)
Supplement: Supplementary file 4 [file Table_2.DOCX]

**Supplementary Table 2. Univariate Cox Regression Models Evaluating the Association Between Lipid Profiles, Their Ratios, and All-Cause Mortality in Patients with Ischemic Stroke at Different Time Points**

| **Variable** | **7-Day** | | | **14-Day** | | | **21-Day** | | | **90-Day** | | |
| --- | --- | --- | --- | --- | --- | --- | --- | --- | --- | --- | --- | --- |
|  | **HR** | **95%CI** | ***P*** | **HR** | **95%CI** | ***P*** | **HR** | **95%CI** | ***P*** | **HR** | **95%CI** | ***P*** |
| **Age** | 1.042 | 1.03 - 1.055 | **< 0.001** | 1.041 | 1.031 - 1.05 | **< 0.001** | 1.040 | 1.032 - 1.049 | **< 0.001** | 1.042 | 1.036 - 1.049 | **< 0.001** |
| **Gender** | 0.622 | 0.469 - 0.825 | **0.001** | 0.709 | 0.568 - 0.884 | **0.001** | 0.730 | 0.596 - 0.894 | **0.002** | 0.770 | 0.657 - 0.903 | **0.001** |
| **Ethnicity: refer. White** |  |  |  |  |  |  |  |  |  |  |  |  |
| **Asian** | 1.237 | 0.577 - 2.651 | 0.585 | 1.182 | 0.644 - 2.169 | 0.589 | 1.088 | 0.609 - 1.943 | 0.776 | 0.880 | 0.534 - 1.451 | 0.617 |
| **Black and Hispanic/Latino** | 0.819 | 0.528 - 1.269 | 0.371 | 0.735 | 0.515 - 1.048 | 0.089 | 0.787 | 0.574 - 1.079 | 0.137 | 0.925 | 0.734 - 1.164 | 0.504 |
| **Other** | 1.897 | 1.366 - 2.633 | **< 0.001** | 1.695 | 1.299 - 2.21 | **< 0.001** | 1.637 | 1.279 - 2.095 | **< 0.001** | 1.420 | 1.158 - 1.74 | **0.001** |
| **Height** | 0.974 | 0.962 - 0.987 | **< 0.001** | 0.979 | 0.969 - 0.989 | **< 0.001** | 0.983 | 0.973 - 0.992 | **< 0.001** | 0.982 | 0.975 - 0.989 | **< 0.001** |
| **Weight** | 0.982 | 0.974 - 0.989 | **< 0.001** | 0.982 | 0.976 - 0.988 | **< 0.001** | 0.983 | 0.977 - 0.988 | **< 0.001** | 0.981 | 0.977 - 0.986 | **< 0.001** |
| **MBP** | 1.002 | 0.991 - 1.013 | 0.720 | 1.000 | 0.992 - 1.009 | 0.974 | 0.999 | 0.991 - 1.007 | 0.815 | 0.990 | 0.984 - 0.996 | **0.002** |
| **SBP** | 1.011 | 1.004 - 1.018 | **0.003** | 1.009 | 1.003 - 1.014 | **0.003** | 1.005 | 1 - 1.011 | **0.041** | 1.000 | 0.996 - 1.004 | 0.902 |
| **DBP** | 0.998 | 0.987 - 1.009 | 0.662 | 0.996 | 0.988 - 1.005 | 0.362 | 0.997 | 0.989 - 1.005 | 0.429 | 0.989 | 0.982 - 0.995 | **< 0.001** |
| **Heart Rate** | 1.013 | 1.004 - 1.022 | **0.004** | 1.016 | 1.009 - 1.023 | **< 0.001** | 1.018 | 1.012 - 1.025 | **< 0.001** | 1.021 | 1.016 - 1.026 | **< 0.001** |
| **Respire Rate** | 1.114 | 1.078 - 1.151 | **< 0.001** | 1.099 | 1.07 - 1.129 | **< 0.001** | 1.097 | 1.07 - 1.125 | **< 0.001** | 1.104 | 1.083 - 1.126 | **< 0.001** |
| **SpO_2_** | 1.034 | 0.959 - 1.116 | 0.383 | 1.058 | 0.995 - 1.125 | 0.072 | 1.061 | 1.003 - 1.123 | **0.041** | 1.048 | 1.002 - 1.096 | **0.039** |
| **GCS** | 0.895 | 0.858 - 0.933 | **< 0.001** | 0.899 | 0.87 - 0.93 | **< 0.001** | 0.900 | 0.872 - 0.928 | **< 0.001** | 0.913 | 0.89 - 0.937 | **< 0.001** |
| **Sofa score** | 1.098 | 1.055 - 1.142 | **< 0.001** | 1.111 | 1.077 - 1.145 | **< 0.001** | 1.127 | 1.097 - 1.158 | **< 0.001** | 1.132 | 1.108 - 1.157 | **< 0.001** |
| **SAPS II** | 1.046 | 1.037 - 1.055 | **< 0.001** | 1.046 | 1.039 - 1.053 | **< 0.001** | 1.048 | 1.041 - 1.054 | **< 0.001** | 1.047 | 1.042 - 1.052 | **< 0.001** |
| **APS III** | 1.026 | 1.021 - 1.032 | **< 0.001** | 1.026 | 1.022 - 1.03 | **< 0.001** | 1.028 | 1.024 - 1.032 | **< 0.001** | 1.028 | 1.024 - 1.031 | **< 0.001** |
| **OASIS** | 1.090 | 1.074 - 1.106 | **< 0.001** | 1.088 | 1.076 - 1.101 | **< 0.001** | 1.089 | 1.077 - 1.101 | **< 0.001** | 1.081 | 1.072 - 1.09 | **< 0.001** |
| **SIRS** | 1.706 | 1.466 - 1.984 | **< 0.001** | 1.707 | 1.515 - 1.924 | **< 0.001** | 1.704 | 1.527 - 1.902 | **< 0.001** | 1.486 | 1.366 - 1.616 | **< 0.001** |
| **Hypertensin** | 1.449 | 0.946 - 2.219 | 0.088 | 1.380 | 0.991 - 1.922 | 0.057 | 1.334 | 0.988 - 1.801 | 0.060 | 1.011 | 0.816 - 1.252 | 0.922 |
| **Diabetes** | 0.751 | 0.554 - 1.017 | 0.064 | 0.737 | 0.579 - 0.938 | **0.013** | 0.799 | 0.643 - 0.994 | **0.044** | 0.943 | 0.798 - 1.113 | 0.486 |
| **Acute Myocardial infarct** | 1.009 | 0.695 - 1.465 | 0.963 | 0.991 | 0.737 - 1.334 | 0.954 | 1.035 | 0.791 - 1.353 | 0.804 | 1.150 | 0.937 - 1.412 | 0.180 |
| **Heart failure** | 1.239 | 0.918 - 1.671 | 0.161 | 1.129 | 0.886 - 1.438 | 0.326 | 1.252 | 1.007 - 1.557 | **0.043** | 1.418 | 1.198 - 1.678 | **< 0.001** |
| **Peripheral vascular disease** | 0.793 | 0.518 - 1.214 | 0.285 | 0.859 | 0.619 - 1.192 | 0.364 | 0.887 | 0.659 - 1.194 | 0.428 | 0.896 | 0.71 - 1.13 | 0.353 |
| **Chronic pulmonary disease** | 0.900 | 0.632 - 1.281 | 0.559 | 0.969 | 0.737 - 1.274 | 0.819 | 1.035 | 0.809 - 1.325 | 0.782 | 1.171 | 0.971 - 1.414 | 0.099 |
| **Respiratory failure** | 1.457 | 1.072 - 1.98 | **0.016** | 1.644 | 1.296 - 2.086 | **< 0.001** | 1.869 | 1.509 - 2.313 | **< 0.001** | 1.731 | 1.458 - 2.055 | **< 0.001** |
| **VAP** | 0.478 | 0.197 - 1.161 | 0.103 | 0.929 | 0.553 - 1.56 | 0.781 | 1.002 | 0.632 - 1.589 | 0.994 | 1.471 | 1.075 - 2.012 | **0.016** |
| **CKD** | 0.894 | 0.63 - 1.268 | 0.530 | 0.930 | 0.707 - 1.223 | 0.602 | 1.077 | 0.846 - 1.371 | 0.548 | 1.331 | 1.111 - 1.596 | **0.002** |
| **Renal failure** | 1.197 | 0.793 - 1.807 | 0.392 | 1.658 | 1.237 - 2.221 | **< 0.001** | 1.934 | 1.497 - 2.5 | **< 0.001** | 2.427 | 2.001 - 2.945 | **< 0.001** |
| **hyperlipidemia** | 0.899 | 0.682 - 1.187 | 0.453 | 0.957 | 0.769 - 1.192 | 0.695 | 0.971 | 0.794 - 1.188 | 0.776 | 0.979 | 0.836 - 1.147 | 0.794 |
| **Malignancy** | 1.293 | 0.815 - 2.052 | 0.276 | 1.621 | 1.155 - 2.274 | **0.005** | 1.907 | 1.42 - 2.561 | **< 0.001** | 2.196 | 1.748 - 2.759 | **< 0.001** |
| **Liver disease** | 0.362 | 0.17 - 0.769 | **0.008** | 0.454 | 0.266 - 0.776 | **0.004** | 0.699 | 0.466 - 1.049 | 0.084 | 1.048 | 0.798 - 1.376 | 0.737 |
| **Sepsis: = 1** | NA | NA | NA | NA | NA | NA | 1.7904 | 0.423 - 7.576 | 0.429 | 0.931 | 0.461 - 1.882 | 0.843 |
| **2** | 3.706 | 1.9 - 7.227 | **< 0.001** | 3.007 | 1.837 - 4.923 | **< 0.001** | 4.3248 | 1.068 - 17.513 | **0.040** | 1.782 | 0.914 - 3.477 | **0.090** |
| **3** | 5.600 | 2.907 - 10.788 | **< 0.001** | 4.905 | 3.035 - 7.927 | **< 0.001** | 7.1741 | 1.779 - 28.936 | **0.006** | 2.509 | 1.29 - 4.882 | **0.007** |
| **4** | 9.001 | 4.536 - 17.859 | **< 0.001** | 7.775 | 4.686 - 12.9 | **< 0.001** | 11.874 | 2.922 - 48.245 | **< 0.001** | 3.826 | 1.941 - 7.54 | **< 0.001** |
| **Charlson comorbidity index** | 1.155 | 1.1 - 1.211 | **< 0.001** | 1.183 | 1.139 - 1.228 | **< 0.001** | 1.204 | 1.164 - 1.246 | **< 0.001** | 1.230 | 1.198 - 1.263 | **< 0.001** |
| **Vasopressors** | 2.643 | 1.682 - 4.154 | **< 0.001** | 2.656 | 1.853 - 3.808 | **< 0.001** | 2.683 | 1.927 - 3.738 | **< 0.001** | 2.805 | 2.153 - 3.654 | **< 0.001** |
| **Oxygen** | 0.376 | 0.285 - 0.496 | **< 0.001** | 0.426 | 0.342 - 0.53 | **< 0.001** | 0.488 | 0.398 - 0.597 | **< 0.001** | 0.732 | 0.62 - 0.865 | **< 0.001** |
| **CRRT** | 1.282 | 0.603 - 2.725 | 0.518 | 1.146 | 0.61 - 2.15 | 0.672 | 1.261 | 0.725 - 2.193 | 0.411 | 1.813 | 1.241 - 2.649 | **0.002** |
| **Thrombolysis** | 0.790 | 0.325 - 1.918 | 0.602 | 0.900 | 0.464 - 1.746 | 0.755 | 0.840 | 0.448 - 1.574 | 0.586 | 1.103 | 0.714 - 1.704 | 0.660 |
| **Thrombectomy** | 0.734 | 0.426 - 1.263 | 0.264 | 1.042 | 0.72 - 1.51 | 0.827 | 1.078 | 0.771 - 1.508 | 0.659 | 1.025 | 0.783 - 1.34 | 0.859 |
| **RBC** | 1.125 | 0.935 - 1.355 | 0.213 | 0.958 | 0.826 - 1.112 | 0.572 | 0.906 | 0.79 - 1.039 | 0.158 | 0.710 | 0.636 - 0.792 | **< 0.001** |
| **WBC** | 1.038 | 1.019 - 1.057 | **< 0.001** | 1.038 | 1.023 - 1.053 | **< 0.001** | 1.043 | 1.03 - 1.056 | **< 0.001** | 1.042 | 1.032 - 1.053 | **< 0.001** |
| **Platelet** | 0.998 | 0.997 - 1 | **0.010** | 0.998 | 0.997 - 0.999 | **0.002** | 0.998 | 0.997 - 0.999 | **0.002** | 0.999 | 0.998 - 1 | **0.036** |
| **Hb** | 1.040 | 0.977 - 1.107 | 0.220 | 0.987 | 0.939 - 1.038 | 0.610 | 0.966 | 0.922 - 1.011 | 0.134 | 0.890 | 0.859 - 0.923 | **< 0.001** |
| **Sodium** | 1.088 | 1.062 - 1.114 | **< 0.001** | 1.085 | 1.063 - 1.106 | **< 0.001** | 1.076 | 1.056 - 1.097 | **< 0.001** | 1.067 | 1.05 - 1.085 | **< 0.001** |
| **Potassium** | 0.963 | 0.759 - 1.222 | 0.757 | 0.938 | 0.775 - 1.136 | 0.512 | 0.905 | 0.757 - 1.081 | 0.270 | 0.899 | 0.781 - 1.035 | 0.138 |
| **BUN** | 1.008 | 1.001 - 1.015 | **0.021** | 1.009 | 1.004 - 1.015 | **0.001** | 1.010 | 1.005 - 1.015 | **< 0.001** | 1.014 | 1.011 - 1.018 | **< 0.001** |
| **Creatinine** | 1.069 | 0.978 - 1.167 | 0.141 | 1.060 | 0.986 - 1.14 | 0.114 | 1.058 | 0.99 - 1.132 | 0.097 | 1.079 | 1.027 - 1.133 | **0.002** |
| **Albumin** | 0.611 | 0.486 - 0.767 | **< 0.001** | 0.530 | 0.444 - 0.633 | **< 0.001** | 0.513 | 0.436 - 0.604 | **< 0.001** | 0.484 | 0.426 - 0.55 | **< 0.001** |
| **ALT** | 1.000 | 1 - 1 | 0.709 | 1.000 | 1 - 1 | 0.680 | 1.000 | 1 - 1 | 0.732 | 1.000 | 1 - 1 | **0.032** |
| **AST** | 1.000 | 1 - 1 | 0.783 | 1.000 | 1 - 1 | 0.750 | 1.000 | 1 - 1 | 0.364 | 1.000 | 1 - 1 | **0.005** |
| **LDH** | 1.000 | 1 - 1 | 0.162 | 1.000 | 1 - 1 | 0.105 | 1.000 | 1 - 1 | 0.114 | 1.000 | 1 - 1 | **0.005** |
| **TC** | 0.996 | 0.994 - 0.999 | **0.008** | 0.997 | 0.995 - 0.999 | **0.002** | 0.996 | 0.995 - 0.998 | **< 0.001** | 0.996 | 0.995 - 0.998 | **< 0.001** |
| **TG** | 0.998 | 0.996 - 0.999 | **0.007** | 0.998 | 0.997 - 0.999 | **< 0.001** | 0.998 | 0.997 - 0.999 | **0.001** | 0.999 | 0.998 - 0.999 | **0.001** |
| **HDL** | 0.999 | 0.991 - 1.006 | 0.706 | 0.997 | 0.991 - 1.003 | 0.323 | 0.997 | 0.991 - 1.002 | 0.254 | 0.995 | 0.991 - 1 | **0.028** |
| **LDL** | 0.997 | 0.993 - 1 | **0.043** | 0.997 | 0.994 - 1 | **0.023** | 0.997 | 0.994 - 0.999 | **0.005** | 0.996 | 0.994 - 0.998 | **< 0.001** |
| **Anion gap** | 1.146 | 1.106 - 1.189 | **< 0.001** | 1.102 | 1.068 - 1.137 | **< 0.001** | 1.092 | 1.061 - 1.125 | **< 0.001** | 1.078 | 1.052 - 1.104 | **< 0.001** |
| **Lactate** | 1.049 | 1.003 - 1.097 | 0.038 | 1.052 | 1.016 - 1.09 | **0.005** | 1.063 | 1.031 - 1.096 | **< 0.001** | 1.075 | 1.05 - 1.101 | **< 0.001** |
| **PT** | 0.991 | 0.969 - 1.013 | 0.407 | 0.988 | 0.97 - 1.007 | 0.207 | 0.995 | 0.98 - 1.01 | 0.523 | 1.007 | 0.999 - 1.016 | 0.105 |
| **APTT** | 0.994 | 0.986 - 1.002 | 0.123 | 0.995 | 0.99 - 1.001 | 0.136 | 0.995 | 0.989 - 1 | 0.062 | 0.997 | 0.993 - 1.001 | 0.193 |
| **INR** | 0.959 | 0.776 - 1.185 | 0.697 | 0.924 | 0.774 - 1.102 | 0.379 | 0.984 | 0.849 - 1.141 | 0.832 | 1.115 | 1.018 - 1.221 | **0.019** |
| **TG/HDL** | 0.987 | 0.954 - 1.02 | 0.426 | 0.982 | 0.955 - 1.01 | 0.201 | 1.000 | 0.981 - 1.02 | 0.992 | 1.003 | 0.987 - 1.018 | 0.742 |
| **TC/HDL** | 0.915 | 0.828 - 1.01 | 0.078 | 0.933 | 0.866 - 1.006 | 0.073 | 0.961 | 0.901 - 1.026 | 0.232 | 0.986 | 0.939 - 1.035 | 0.568 |
| **LDL/HDL** | 0.886 | 0.773 - 1.016 | 0.084 | 0.918 | 0.827 - 1.019 | 0.107 | 0.929 | 0.845 - 1.021 | 0.126 | 0.966 | 0.899 - 1.038 | 0.343 |
| **WBC/HDL** | 1.863 | 1.173 - 2.958 | **0.008** | 1.937 | 1.364 - 2.75 | **< 0.001** | 2.304 | 1.75 - 3.034 | **< 0.001** | 2.516 | 2.01 - 3.15 | **< 0.001** |
