# Supplementary material for: The WBC/HDL ratio outperforms other lipid profiles in predicting mortality among ischemic stroke patients: a retrospective cohort study using MIMIC-IV data
Source: Front Neurol. 2025 Apr 30;16:1534381. doi: 10.3389/fneur.2025.1534381 (PMC12074928; doi:10.3389/fneur.2025.1534381)
Supplement: Supplementary file 5 [file Table_3.DOCX]

**Supplementary Table 3. Results of collinearity test of initial models in multi-model Cox regression analysis**

| **Model*** | **Variable** | **28-Day** | | **1-Year** | |
| --- | --- | --- | --- | --- | --- |
|  |  | **Coefficient** | **VIF** | **Coefficient** | **VIF** |
| **Model_0** | **TC** | -0.006 | **17.501** | -0.002 | **12.795** |
|  | **TG** | -0.001 | 1.739 | -0.001 | 1.758 |
|  | **LDL** | 0.005 | **12.380** | -0.001 | **9.012** |
|  | **HDL** | 0.006 | 2.975 | 0.002 | 2.505 |
|  | **WBC/HDL** | 0.976 | 1.401 | 0.808 | 1.364 |
| **Model_1** | **Age** | 0.042 | 1.128 | 0.041 | 1.107 |
|  | **Gender** | -0.110 | 1.107 | -0.132 | 1.100 |
|  | **TC** | -0.006 | **18.168** | -0.002 | **13.001** |
|  | **TG** | -0.001 | 1.773 | 0.000 | 1.809 |
|  | **LDL** | 0.006 | **12.827** | 0.001 | **9.151** |
|  | **HDL** | 0.001 | 2.969 | -0.002 | 2.510 |
|  | **WBC/HDL** | 1.122 | 1.444 | 0.948 | 1.400 |
| **Model_2** | **Age** | 0.028 | 1.347 | 0.027 | 1.331 |
|  | **Gender** | -0.126 | 1.100 | -0.144 | 1.090 |
|  | **CCI** | 0.135 | 1.239 | 0.144 | 1.247 |
|  | **TC** | -0.006 | **17.127** | -0.002 | **12.762** |
|  | **TG** | -0.001 | 1.712 | 0.000 | 1.776 |
|  | **LDL** | 0.007 | **12.311** | 0.002 | **9.129** |
|  | **HDL** | 0.002 | 2.819 | -0.002 | 2.445 |
|  | **WBC/HDL** | 1.010 | 1.460 | 0.820 | 1.414 |
| **Model_3** | **Age** | 0.041 | 1.130 | 0.040 | 1.103 |
|  | **Gender** | -0.179 | 1.108 | -0.202 | 1.103 |
|  | **SOFA Score** | 0.121 | 1.107 | 0.116 | 1.089 |
|  | **TC** | -0.005 | **17.896** | -0.001 | **13.179** |
|  | **TG** | -0.001 | 1.892 | -0.001 | 1.958 |
|  | **LDL** | 0.006 | **12.432** | 0.001 | **9.086** |
|  | **HDL** | -0.001 | 2.952 | -0.004 | 2.515 |
|  | **WBC/HDL** | 0.884 | 1.499 | 0.730 | 1.427 |
| **Model_4** | **Age** | 0.031 | 1.371 | 0.029 | 1.341 |
|  | **Gender** | -0.224 | 1.119 | -0.230 | 1.110 |
|  | **CCI** | 0.119 | 1.240 | 0.130 | 1.244 |
|  | **SOFA Score** | 0.089 | 1.357 | 0.089 | 1.294 |
|  | **Vasopressors** | 0.722 | 1.291 | 0.580 | 1.231 |
|  | **TC** | -0.004 | **17.595** | -0.001 | **13.122** |
|  | **TG** | -0.001 | 1.846 | -0.001 | 1.906 |
|  | **LDL** | 0.006 | **12.486** | 0.001 | **9.227** |
|  | **HDL** | -0.001 | 2.877 | -0.004 | 2.467 |
|  | **WBC/HDL** | 0.768 | 1.520 | 0.615 | 1.435 |
| **Model_5** | **Age** | 0.031 | 1.375 | 0.029 | 1.345 |
|  | **Gender** | -0.234 | 1.130 | -0.233 | 1.118 |
|  | **CCI** | 0.120 | 1.243 | 0.131 | 1.246 |
|  | **SOFA Score** | 0.083 | 1.463 | 0.081 | 1.377 |
|  | **Vasopressors** | 0.676 | 1.434 | 0.467 | 1.357 |
|  | **Platelet** | 0.000 | 1.059 | 0.000 | 1.055 |
|  | **Lactate** | 0.019 | 1.384 | 0.040 | 1.309 |
|  | **TC** | -0.004 | **17.854** | -0.001 | **13.315** |
|  | **TG** | -0.001 | 1.875 | -0.001 | 1.932 |
|  | **LDL** | 0.006 | **12.685** | 0.001 | **9.367** |
|  | **HDL** | -0.001 | 2.909 | -0.004 | 2.495 |
|  | **WBC/HDL** | 0.741 | 1.562 | 0.565 | 1.463 |

*: The original models, Model 0: only lipids and lipid profiles; Model 1: main variables of interest + basic demographic variables; Model 2: main variables of interest + basic demographic variables + Charlson Comorbidity Index; Model 3: main variables of interest + basic demographic variables + SOFA Score; Model 4: comprehensive model 1; Model 5: comprehensive model 2 + incorporating important laboratory tests
