# Supplementary material for: The WBC/HDL ratio outperforms other lipid profiles in predicting mortality among ischemic stroke patients: a retrospective cohort study using MIMIC-IV data
Source: Front Neurol. 2025 Apr 30;16:1534381. doi: 10.3389/fneur.2025.1534381 (PMC12074928; doi:10.3389/fneur.2025.1534381)
Supplement: Supplementary file 6 [file Table_4.DOCX]

**Supplementary Table 4. Results of collinearity test of adjusted models in multi-model Cox regression analysis**

| **Model^#^** | **Variable** | **28-Day** | | **1-Year** | |
| --- | --- | --- | --- | --- | --- |
|  |  | **Coefficient** | **VIF** | **Coefficient** | **VIF** |
| **Model_0** | **TG** | -0.002 | 1.050 | -0.001 | 1.031 |
|  | **HDL** | 0.000 | 1.341 | -0.001 | 1.343 |
|  | **WBC/HDL** | 1.003 | 1.349 | 0.867 | 1.333 |
| **Model_1** | **Age** | 0.042 | 1.118 | 0.041 | 1.095 |
|  | **Gender** | -0.102 | 1.104 | -0.126 | 1.096 |
|  | **TG** | -0.001 | 1.068 | 0.000 | 1.053 |
|  | **HDL** | -0.003 | 1.457 | -0.005 | 1.456 |
|  | **WBC/HDL** | 1.117 | 1.396 | 0.972 | 1.372 |
| **Model_2** | **Age** | 0.028 | 1.345 | 0.027 | 1.325 |
|  | **Gender** | -0.123 | 1.099 | -0.140 | 1.088 |
|  | **CCI** | 0.134 | 1.239 | 0.145 | 1.240 |
|  | **TG** | -0.001 | 1.068 | 0.000 | 1.053 |
|  | **HDL** | -0.002 | 1.470 | -0.004 | 1.460 |
|  | **WBC/HDL** | 0.984 | 1.421 | 0.822 | 1.392 |
| **Model_3** | **Age** | 0.040 | 1.122 | 0.040 | 1.094 |
|  | **Gender** | -0.177 | 1.107 | -0.200 | 1.102 |
|  | **SOFA Score** | 0.121 | 1.092 | 0.116 | 1.076 |
|  | **TG** | -0.002 | 1.087 | -0.001 | 1.071 |
|  | **HDL** | -0.004 | 1.448 | -0.005 | 1.442 |
|  | **WBC/HDL** | 0.854 | 1.456 | 0.734 | 1.404 |
| **Model_4** | **Age** | 0.030 | 1.372 | 0.029 | 1.337 |
|  | **Gender** | -0.223 | 1.118 | -0.229 | 1.109 |
|  | **CCI** | 0.118 | 1.240 | 0.130 | 1.240 |
|  | **SOFA Score** | 0.088 | 1.346 | 0.089 | 1.283 |
|  | **Vasopressors** | 0.719 | 1.293 | 0.579 | 1.230 |
|  | **TG** | -0.002 | 1.092 | -0.001 | 1.074 |
|  | **HDL** | -0.003 | 1.463 | -0.004 | 1.446 |
|  | **WBC/HDL** | 0.723 | 1.481 | 0.604 | 1.416 |
| **Model_5** | **Age** | 0.030 | 1.376 | 0.029 | 1.342 |
|  | **Gender** | -0.232 | 1.129 | -0.232 | 1.117 |
|  | **CCI** | 0.119 | 1.244 | 0.131 | 1.242 |
|  | **SOFA Score** | 0.082 | 1.453 | 0.080 | 1.368 |
|  | **Vasopressors** | 0.669 | 1.436 | 0.466 | 1.355 |
|  | **Platelet** | 0.000 | 1.052 | 0.000 | 1.048 |
|  | **Lactate** | 0.020 | 1.391 | 0.040 | 1.309 |
|  | **TG** | -0.002 | 1.108 | -0.001 | 1.086 |
|  | **HDL** | -0.003 | 1.468 | -0.005 | 1.450 |
|  | **WBC/HDL** | 0.693 | 1.525 | 0.553 | 1.439 |

#: The adjusted model removes the variable with a large VIF
